# Inhibition of gap junction composed of Cx43 prevents against acute kidney injury following liver transplantation

**DOI:** 10.1038/s41419-019-1998-y

**Published:** 2019-10-10

**Authors:** Dongdong Yuan, Xiaoyun Li, Chenfang Luo, Xianlong Li, Nan Cheng, Haocong Ji, Rongzong Qiu, Gangjian Luo, Chaojin Chen, Ziqing Hei

**Affiliations:** 1 Department of Anesthesiology, the third affiliated Hospital of Sun Yat-sen University, Tianhe Road, Guangzhou, PR China; 2 Department of Anesthesiology, Huizhou first People’s Hospital, No. 20, San Xin Nan Road, Jiangbei, Huizhou PR China

**Keywords:** Acute kidney injury, Translational research

## Abstract

Postoperative acute kidney injury (AKI) is a severe complication after liver transplantation (LT). Its deterioration and magnification lead to the increase in mortality. Connexin43 (Cx43) mediates direct transmission of intracellular signals between neighboring cells, always considered to be the potent biological basis of organ damage deterioration and magnification. Thus, we explored the effects of Cx43 on AKI following LT and its related possible mechanism. In this study, alternations of Cx43 expression were observed in 82 patients, receiving the first-time orthotopic LT. We built autologous orthotopic liver transplantation (AOLT) models with Sprague–Dawley (SD) rats in vivo, and hypoxia-reoxygenation (H/R) or lipopolysaccharide (LPS) pretreatment models with kidney tubular epithelial cells (NRK-52E) in vitro, both of which were the most important independent risk factors of AKI following LT. Then, different methods were used to alter the function of Cx43 channels to determine its protective effects on AKI. The results indicated that patients with AKI suffering from longer time of tracheal intubation or intensive care unit stay, importantly, had significantly lower survival rate at postoperative 30 days and 3 years. In rat AOLT models, as Cx43 was inhibited with heptanol, postoperative AKI was attenuated significantly. In vitro experiments, downregulation of Cx43 with selective inhibitors, or siRNA protected against post-hypoxic NRK-52E cell injuries caused by H/R and/or LPS, while upregulation of Cx43 exacerbated the above-mentioned cell injuries. Of note, alternation of Cx43 function regulated the content of reactive oxygen species (ROS), which not only mediated oxidative stress and inflammation reactions effectively, but also regulated necroptosis. Therefore, we concluded that Cx43 inhibition protected against AKI following LT through attenuating ROS transmission between the neighboring cells. ROS alternation depressed oxidative stress and inflammation reaction, which ultimately reduced necroptosis. This might offer new insights for targeted intervention for organ protection in LT, or even in other major surgeries.

## Introduction

Liver transplantation (LT) is the most effective therapy for patients with end-stage liver disease^1^. However, the operation is a huge trauma to patients that usually results in severe complications perioperatively. Postoperative AKI is one of the most severe complications, which not only delays the recovery of patients, but also decreases the survival rate^2,3^. The causes of postoperative AKI are complicated and involve multiple factors, among which renal hypoperfusion induced by hypotension and renal toxicity mediated by endotoxins are considered to be two of the most important independent risk factors^4^. During LT, both the inferior vena cava and portal vein are interrupted, which inevitably induce hypotension and intestinal congestion. Hypotension would cause renal hypoperfusion-induced ischemia–reperfusion (I/R) injury, while intestinal congestion leads to endotoxin production^5,6^. These events increase the risk of renal, oxidative, and inflammatory injury. If oxidative stress and inflammatory reaction are not controlled effectively, renal injury would continue to magnify and deteriorate, which eventually exerts serious impacts on the prognosis of LT patients. However, the detailed mechanism involved in this pathology is poorly known.

Gap junctions (GJs) mediate direct cell-to-cell transfer of molecules and/or electrical charge^7^, always considered to be the potent biological basis of organ damage deterioration and magnification. Thus, we explored its effects on AKI following LT and its related possible mechanism. GJs are composed of connexin proteins. Six connexin monomers form a hemichannel, and then dock to a counterpart of the neighboring cell to form an integral GJ. Gap junctional intercellular communication (GJIC) is crucial for cell differentiation and growth, normal physiology, and regulation of oxidative stress and inflammation reaction in different organs^8^. Thus, the roles of GJ and connexin in organ protection against injury have attracted considerable interest^9^. At present, 21 isoforms of connexin have been identified, almost in all human organs and tissues, each of which has distinct regulation and permeability corresponding to different functions^10,11^. Cx43 is widely expressed in human organs. It had been reported that Cx43 inhibition protected the brain or myocardium against I/R injuries through attenuating oxidative stress and cell apoptosis^12,13^, and death signal transduction mediated by Cx43 could lead to the continuous expansion of injury^14^, which prompts us that GJ composed of Cx43 mediating the direct molecules transfer between the neighboring cells might be responsible for the renal damage deterioration and magnification.

Although the study about the intrinsic quality of direct molecules transfer has been going on for some time, it is still controversial. ROS, including oxygen radicals and nonradical compounds, is but one of the signals that could be transmitted through Cx43 channels. Multiple studies demonstrated that ROS was always hypothesized as the “motor” of oxidative stress and inflammation reaction^15^. With the fact that Cx43 expression in kidneys was increased significantly in this study, we speculated that GJ composed of Cx43 mediated direct ROS transfer between the neighboring cells, which initiated oxidative stress and inflammation reaction, and resulted in renal injuries magnified and deteriorated. According to the reports, severe oxidative stress and inflammation reaction always results in necroptosis^16,17^, which prompts us to consider whether AKI induced by LT is relative with necroptosis.

Thus, in this study, we first analyzed the effects of AKI followed by LT on patients and found that postoperative AKI significantly affected patients’ recovery and survival rate. Hypotension and endotoxin production, two of the most important independent risk factors of postoperative AKI, were also increased obviously. Second, we found that in rat AOLT models, increase in kidney Cx43 expression correlated with the most severe functional impairment and pathological damage after AOLT. Thus, different methods with totally distinct mechanisms were used to change Cx43 function in rat AOLT models and in NRK-52E cells exposed to H/R and/or LPS to test the hypothesis that Cx43 might play an important role in AKI following LT. Finally, we explored the possible mechanisms of Cx43 protecting against renal damage following LT, which was relative with ROS transfer between the neighboring cells through GJ composed of Cx43. The changes in ROS-regulated oxidative stress and inflammation reaction in kidneys, ultimately, influenced necroptosis.

## Results

### LT-induced AKI delayed patients’ recovery and reduced survival rate

**Fig. 1:**
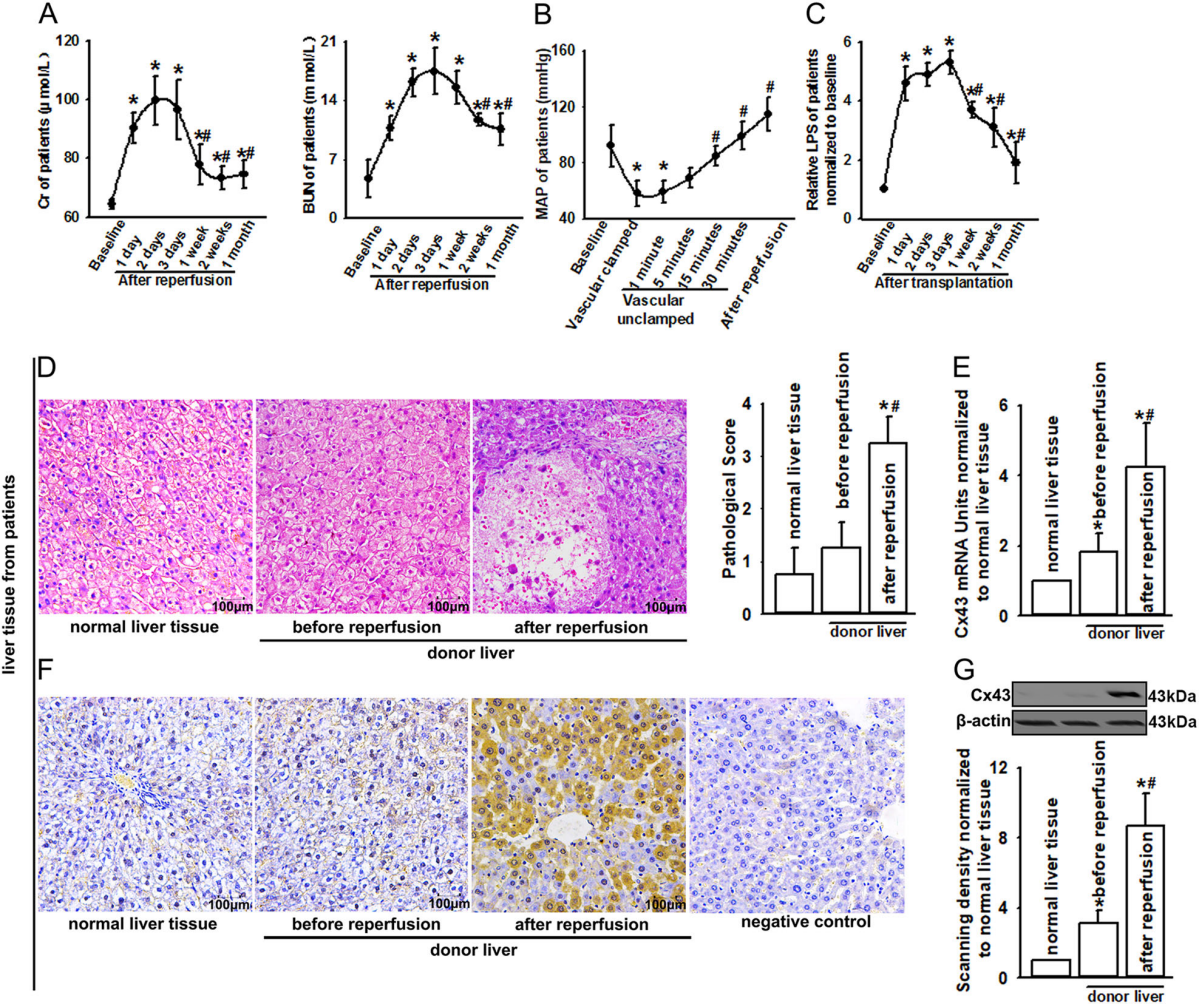
Effects of LT on patients. **a** Changes in patients’ Cr and BUN, at different reperfusion time points after LT. *n* = 38 (patients with AKI after LT), *P < 0.05 vs patient baseline before LT; ^#^*P* < 0.05 vs 3 days after reperfusion. **b** Changes in patients’ MAP during operation. *n* = 38 (patients with AKI after LT), *P < 0.05 vs patient baseline before LT; ^#^*P* < 0.05 vs vascular clamped. **c** Changes in patients’ LPS at different reperfusion time points after LT. *n* = 38 (patients with AKI after LT), *P < 0.05 vs patient baseline before LT. **d** Donor liver damage of patients after LT (H&E staining; original magnification ×200) and pathological scores. **e**–**g** The level of Cx43 mRNA (qPCR) and Cx43 expression alternation after LT (immunohistochemical staining and western blotting; original magnification ×200). Tissues of donor liver were obtained from eight patients before or after reperfusion during the trimming phase, and normal liver tissues were obtained from eight patients with hepatic hemangioma

Table 1 shows the details of the major clinical characteristics of patients. Thirty-eight patients experienced AKI. Renal functional injury, manifested as increased levels of serum Cr and BUN, deteriorated gradually and peaked at 2 or 3 days after reperfusion, and then recovered (Fig. 1a). Although the duration of hospital stay did not differ significantly, patients with AKI suffered from significantly longer time with a respirator and staying in the intensive care unit, and had significantly lower survival rate after 30 days, and 1–3 years than patients without AKI. The 3-year survival rate of patients with AKI was 53.2% compared with 81.7% in patients without AKI.

**Table 1:** Characteristics of 82 patients after liver transplantation

	With AKI	Without AKI	Z	*P*
Patient number	38	44		
Respirator time (h)	65 (38.25, 77)	19.25 (10.125, 30.125)	−6.376	<0.001
Intensive care unit residence time (h)	249.5 (183, 345)	92.5 (65, 195.25)	−3.990	<0.001
Hospital time (day)	55.5 (45, 71.25)	54 (43, 65)	−0.214	0.830
Survival rate at 30 days after operation	86.8%	100.0%*		
Survival rate at 1 year of operation	70.9%	95.2%*		
Survival rate at 2 years of operation	61.1%	90.5%*		
Survival rate at 3 years of operation	53.2%	81.7%*		

Characteristics of patients with or without AKI after liver transplantation. Respirator time, intensive care unit residence time, and hospital time are present, all as median with interquartile range. Compared with the AKI group, *P < 0.05

### Patients receiving LT experienced prolonged hypotension and serious endotoxemia

As illustrated in Fig. 1b, patients with postoperative AKI experienced prolonged hypotension when the inferior vena cava and portal vein were clamped during operation, and the MAP did not recover to the baseline value until 30 min after vascular unclamping. Also, LPS production was gradually and significantly increased from 4 h after reperfusion, and maintained at high levels for over 2 weeks after surgery (Fig. 1c). Compared with patients without AKI, patients with AKI experienced more severe hypotension and endotoxemia. Supplementary Fig. 1A showed that patients with AKI experienced serious and long-term hypotension compared with those without AKI, and more importantly, the level of MAP was <60 mmHg for ~10 min, which is always considered to be one of the most important factors resulting in AKI^18^. Similar to the alternation of MAP, patients with AKI experienced more severe endotoxemia. Especially in the first 3 days after LT, the levels of LPS in patients with AKI were higher than those without AKI (Supplementary Fig. 1B).

### Cx43 expression was increased in donor livers of patients and in rats

**Fig. 2:**
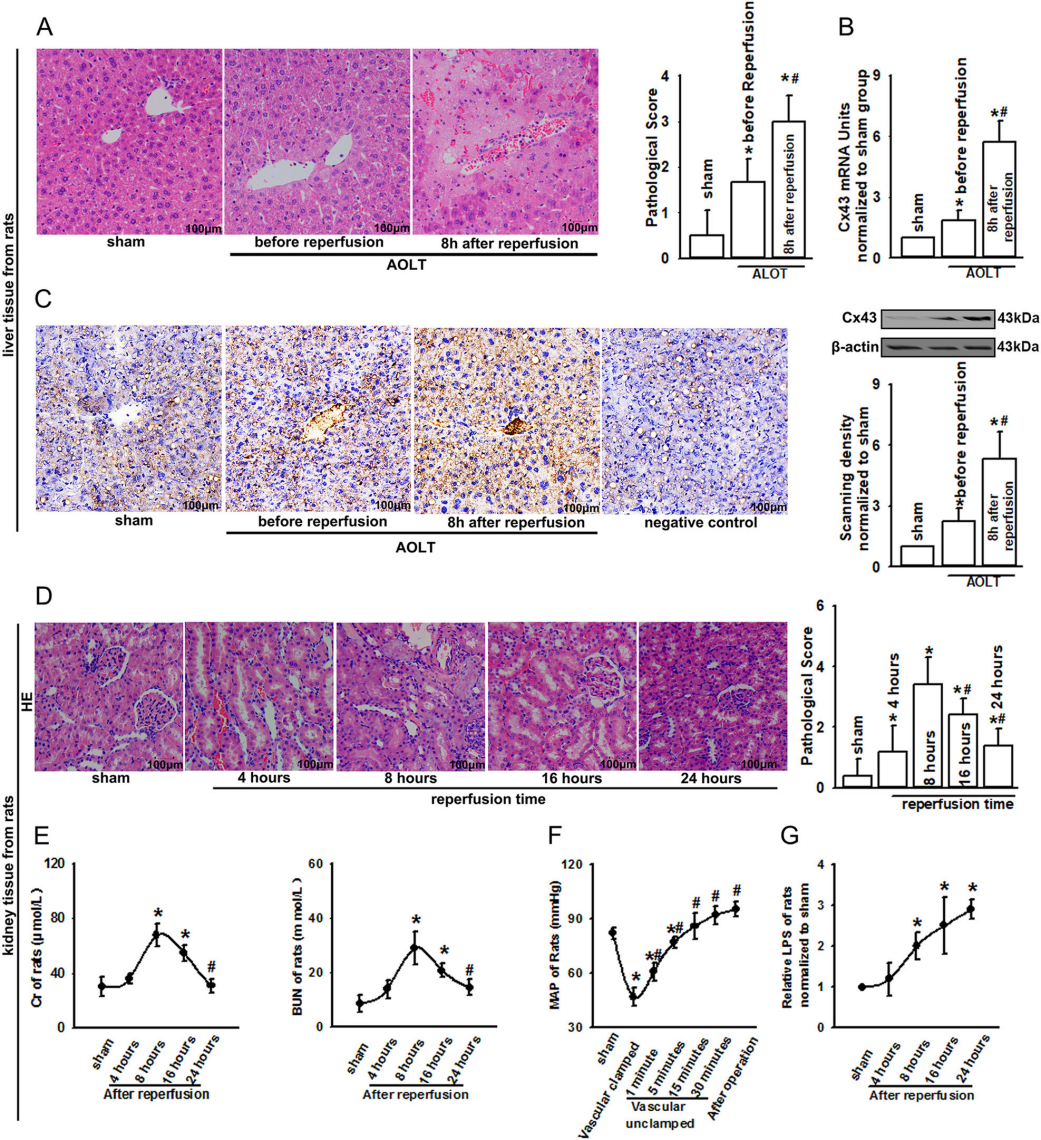
Effects of AOLT on rats. **a** Liver damage of rats after AOLT (H&E staining; original magnification ×200). **b**, **c** The level of Cx43 mRNA (qPCR) and Cx43 expression alternation after LT (immunohistochemical staining and western blotting; original magnification ×200). **d** Kidney damage of rats after AOLT at different reperfusion time points (H&E staining; original magnification ×200). **e** Changes in rat Cr and BUN at different reperfusion time points after AOLT. *n* = 8, *P < 0.05 vs Sham; ^#^*P* < 0.05 vs 8 h after reperfusion. **f** Changes in MAP at different reperfusion time points after AOLT. *n* = 8, *P < 0.05 vs Sham; ^#^*P* < 0.05 vs vascular clamped. **g** Changes in LPS at different reperfusion time points after AOLT. *n* = 8, *P < 0.05 vs Sham

The acquisition of kidney tissue from patients undergoing LT is almost impossible. However, we noticed that donor liver experienced the similar pathophysiological process as kidneys, such as prolonged hypotension and serious endotoxemia. Thus, donor liver tissues of patients were obtained to explore the effects of Cx43 on organ damage. As shown in Fig. 1d, donor liver displayed more severe lobular distortion with necrosis, apparent edema, hemorrhage, and neutrophil infiltration after reperfusion, compared with donor liver tissues obtained before reperfusion or normal liver tissue (from patients with hepatic hemangioma). After reperfusion, the level of Cx43 mRNA (detected by qPCR, Fig. 1e) and Cx43 protein expression (detected by both western blotting and immunostaining, Fig. 1f, g) in donor liver were significantly higher than that before reperfusion or normal liver tissue, which was coincident with more severe liver pathological damage. Of note, both liver damage and Cx43 expression of rats receiving AOLT were similar to that of patients undergoing LT. Compared with sham group or samples obtained before reperfusion, hepatic tissue demonstrated more severe lobular distortion with necrosis and apparent edema 8 h after reperfusion (Fig. 2a). Moreover, the level of Cx43 mRNA and Cx43 expression in rat hepatic tissue were significantly higher than that before reperfusion or sham groups (Fig. 2b, c). In all of the results above, we found that damages of organs experiencing hypotension or endotoxemia were always accompanied with Cx43 expression increase.

### Prolonged hypotension, endotoxin production, and renal injury in rat AOLT models were similar to that in patients undergoing LT

To directly explore whether AKI following LT was relative with Cx43 expression alternation, well-established rat AOLT models were used in our investigation. Figure 2d shows that the kidney sections from the sham group revealed the normal structure and tubular epithelial cells without signs of degeneration or necrosis. As reperfusion time being extended, kidney pathological damage became more serious, and reached to the peak at 8 h after reperfusion: kidney tissue displayed the most serious tubular damage as evidenced by tubular dilatation, vacuolation, loss of the brush border, tubular necrosis, and peritubular capillary congestion. After that, it was recovered gradually. The same trend was also found in the changes of Cr and BUN (Fig. 2e). When portal vein (PV), supra hepatic vena cava (SVC), inferior vena cava (IVC), and hepatic artery were all clamped, rat MAP decreased dramatically, and it did not restore to the normal level until 30 min after vascular unclamped (Fig. 2f). Post-AOLT LPS production increased significantly and had the same trend as that of patients (Fig. 2g). In rat AOLT models, both prolonged hypotension and endotoxin production mirrored the patterns of hypotension and endotoxin production seen in patients undergoing LT.

### Inhibition of Cx43 attenuated AKI following AOLT in rats

**Fig. 3:**
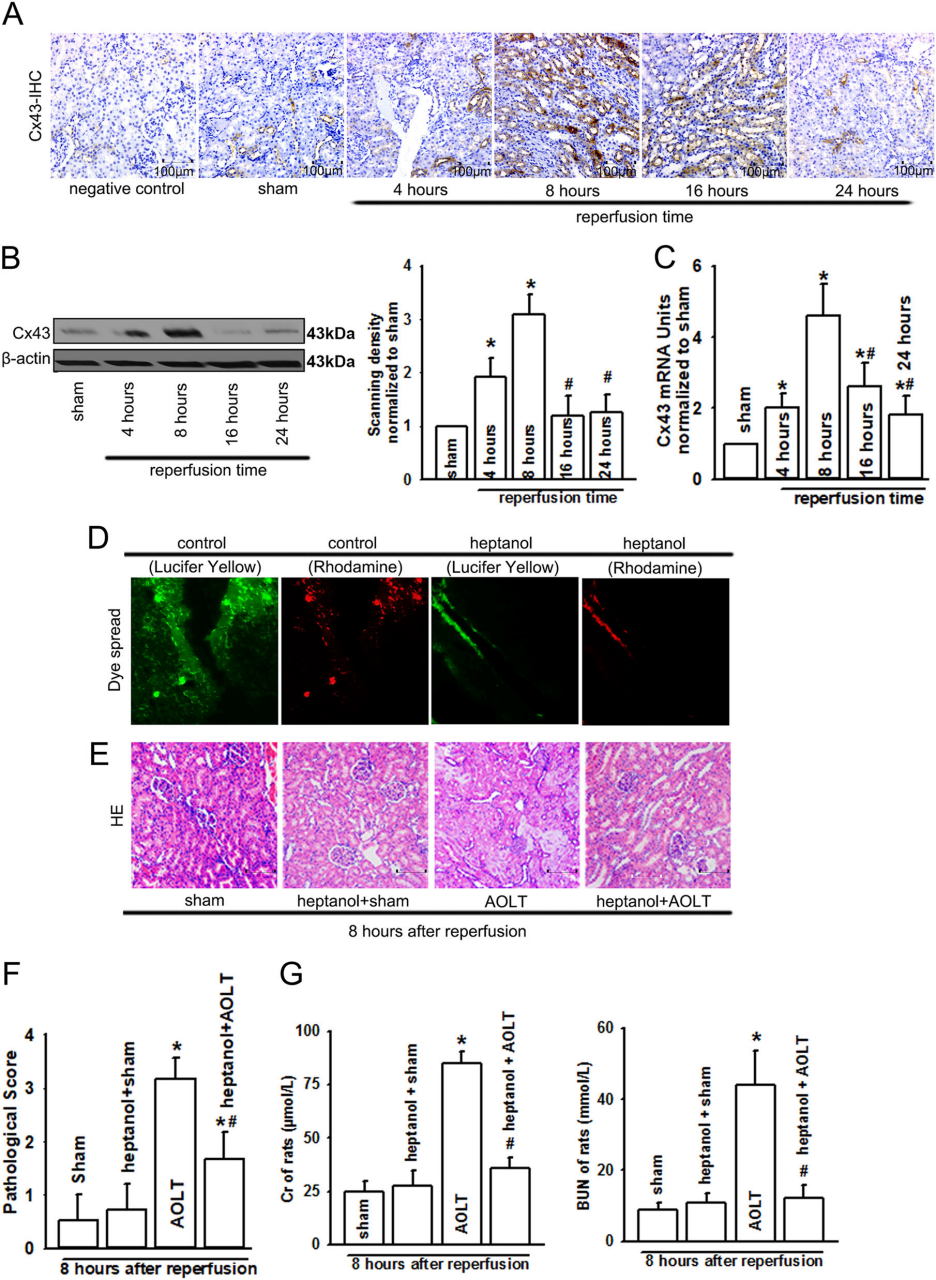
Kidney damage following AOLT was attenuated by inhibiting Cx43 function in vivo. **a**–**c** The level of Cx43 mRNA (qPCR) and Cx43 expression alternation (immunohistochemical staining and western blotting; original magnification ×200) in rat kidneys at different reperfusion time points after AOLT. *n* = 8, *P *<* 0.05 vs sham; ^#^*P* < 0.05 vs 8 h after reperfusion. **d** “Scrape-and-load” assay was used to evaluate functional Cx43 in kidney tissue. Rats were treated with heptanol (0.1 mg/kg) or the corresponding solvent, DMSO for 1 h. The function of Cx43 was demonstrated by the spread of GJ-permeable Lucifer yellow. Rhodamine was impermeable as a negative control. DMSO had no effects on the results (data not shown), *n* = 6. **e**, **f** Remote kidney damage at 8 h after reperfusion, when rats were exposed to heptanol (0.1 mg/kg) or DMSO for 1 h before AOLT (H&E staining; original magnification ×200 ). DMSO had no effects on the results (data not shown), *n* = 8. **g** Changes in Cr and BUN at 8 h after reperfusion, when rats were exposed to heptanol (0.1 mg/kg) or DMSO for 1 h before AOLT. DMSO had no effects on the results (data not shown). *n* = 8, *P < 0.05 vs sham; ^#^*P* < 0.05 vs the AOLT group

Cx43 participates in different organ injuries^19–21^. Increases in Cx43 expression in liver tissues either from patients or rats were coincident with its pathological damage (Figs. 1d–g, 2a, b). We then analyzed the expression of Cx43 in rats’ kidney following AOLT. As shown in Fig. 3a–c, Cx43 expression increased dramatically at 8 h after reperfusion, which was consistent with renal functional impairment and pathological damage as shown in Fig. 2c, d. Moreover, when NRK-52E cells were exposed to hypoxia for 24 h and reoxygenation for 4 h (H24R4), LPS, or the combination of H24R4 and LPS, Cx43 expression was also increased significantly (Supplementary Fig. 2). From the results we obtained, we speculated that the increase in Cx43 expression might play an important role in AKI following AOLT. To explore the hypothesis, we used heptanol, a well-known inhibitor of Cx43 without hepatotoxicity as shown by others^22^ and our preliminary data (Supplementary Figs. 3, 4), to alter the function of GJ composed of Cx43 (Supplementary Fig. 3C, D also showed that heptanol had no effects on Cx43 expression in kidneys or livers. Moreover, Supplementary Table 1 and Supplementary Fig. 3E showed that heptanol has no effects on hypotension and endotoxemia). “Scrape-and-load” assay confirmed that heptanol effectively blocked Cx43 channel function, manifested as the reduction of dye spread (Lucifer Yellow) on kidney slices, and Rhodamine was not influenced by heptanol (Lucifer Yellow could be transferred through GJ, and Rhodamine was impermeable) (Fig. 3d). As the function of Cx43 channels was inhibited with heptanol significantly, the severity of tubular injury was also reduced (Fig. 3e). The structure of most kidney tubular cells was normal, and few of tubular dilatation, vacuolation, brush border loss, tubular necrosis, or peritubular capillary congestion were observed (Fig. 3f). Meanwhile, Cx43 channel function inhibition also improved the levels of Cr and BUN (Fig. 3g).

### Cx43 channel function alternation affected H24R4 or LPS-induced NRK-52E cell injury

**Fig. 4:**
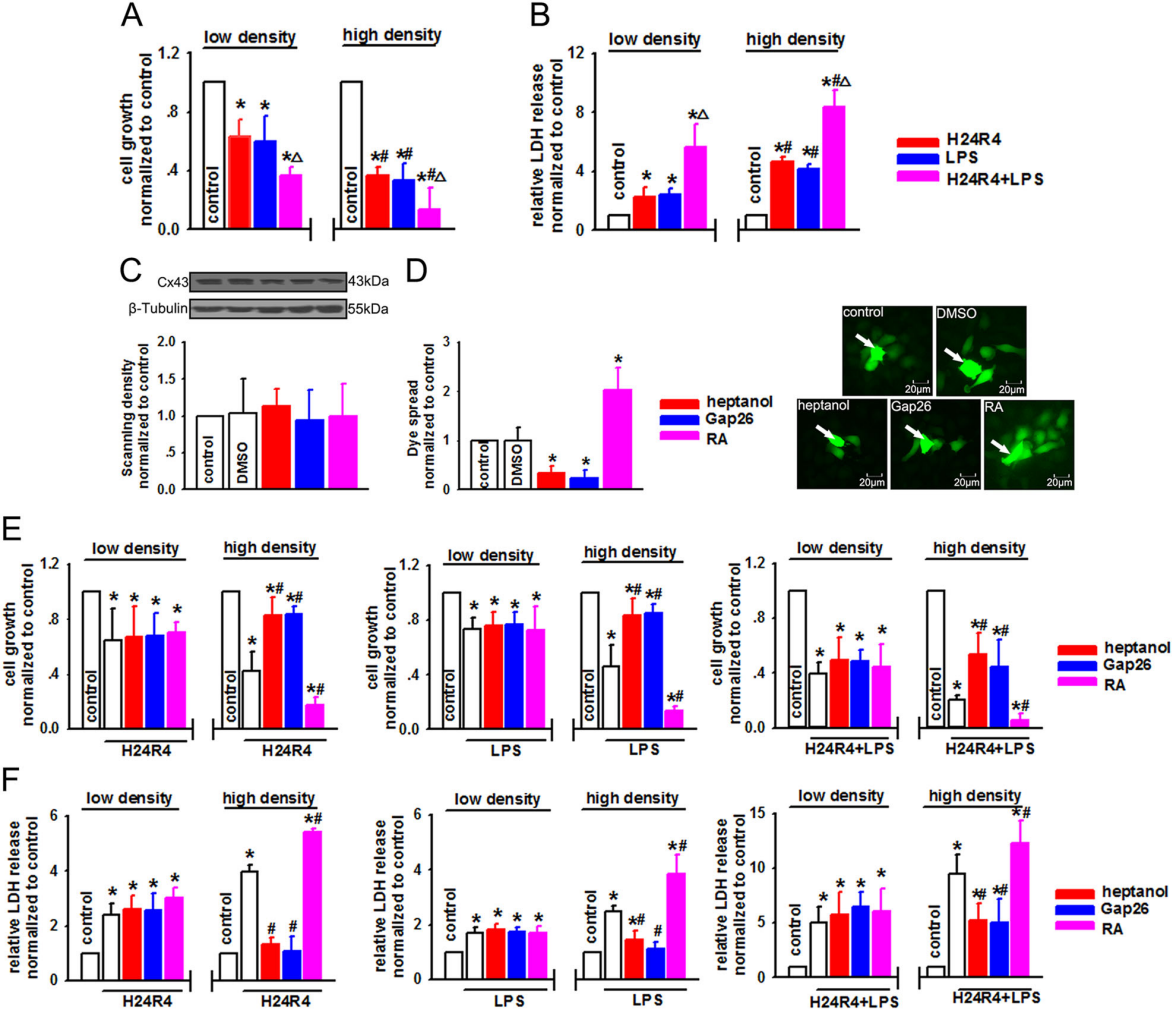
NRK-52E cells damage induced by H24R4 or/and LPS was regulated by the function of Cx43 channels. **a**, **b** NRK-52E cells growth at low- and high-density cell culture exposed to H24R4, LPS (5 μg/ml, 24 h), or a combination of H24R4 and LPS. *n* = 6, *P < 0.05 vs control; ^#^*P* < 0.05 vs the same treatment groups at low-density cell culture; △*P* < 0.05 vs the respective treatment of H24R4 or LPS. **c**, **d** Heptanol (2 mM, 1 h), Gap26 (300 μM, 1 h), and RA (10 μM, 24 h) had no effects on Cx43 expression in NRK-52E cells, but changed dye coupling. *n* = 5, *P < 0.05 vs control. The arrows were the donor cells. **e** Effects of heptanol (2 mM, 1 h), Gap26 (300 μM, 1 h), and RA (10 μM, 24 h) on cell growth at low- and high-density cell culture exposed to H24R4, LPS (5 μg/ml, 24 h), or a combination of H24R4 and LPS. *n* = 6, *P < 0.05 vs control; ^#^*P* < 0.05 vs the same treatment groups at low-density cell culture. **f** Effects of heptanol (2 mM, 1 h), Gap26 (300 μM, 1 h), and RA (10 μM, 24 h) on LDH release at low- and high-density cell culture exposed to H24R4, LPS (5 μg/ml, 24 h), or a combination of H24R4 and LPS. *n* = 6, *P < 0.05 vs control; ^#^*P* < 0.05 vs the same treatment groups at low-density cell culture

**Fig. 5:**
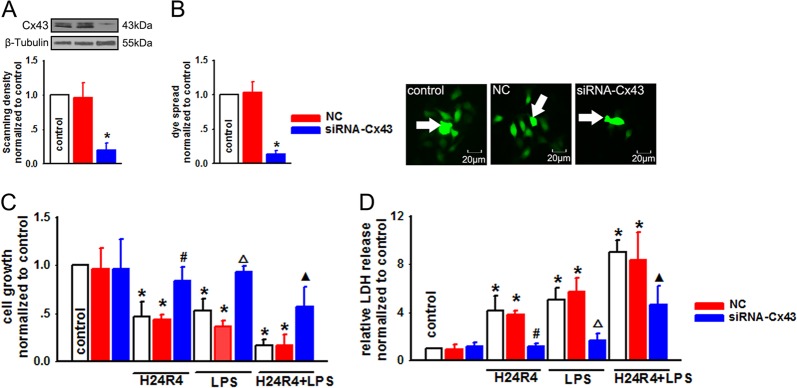
SiRNA modulated the function of Cx43 channels and attenuated NRK-52E cells damage caused by H24R4, LPS, or a combination of H24R4 and LPS. **a**, **b** Specific siRNA–Cx43 downregulated Cx43 expression and function. *n* = 5, *P < 0.05 vs control. The arrows were the donor cells. **c**, **d** Specific siRNA increased NRK-52E cell growth, but decreased LDH release when exposed to H24R4, LPS (5 μg/ml, 24 h), or a combination of H24R4 and LPS. *n* = 5, *P < 0.05 vs control; ^#^*P* < 0.05 vs the H24R4 group; △*P* < 0.05 vs the LPS group; ▲*P* < 0.05 vs H24R4 + LPS group

In addition to I/R injury, renal toxicity induced by endotoxins is another independent risk factor for AKI following LT^23^. We then exposed NRK-52E cells to H/R without or with concomitant LPS to study the effects of Cx43 on renal toxicity induced by H/R and endotoxin. Three different methods with totally distinct mechanisms were employed to alter GJ function composed of Cx43 in NRK-52E cells. First, NRK-52E cells were seeded at low-density (25,000 cells/cm^2^, no GJ formed) and high-density cell culture (125,000 cells/cm^2^, GJ formed), respectively. Figure 4a shows that when being exposed to H24R4, LPS, or a combination of the two risk factors, NRK-52E cell growth declined at both low-density and high-density cell culture. After being subjected to the combination of H24R4 and LPS, cell growth was significantly further reduced. Interestingly, when cells were cultured at high density, cell damage was more striking than that seen at low-density culture. Likewise, when GJ was formed at high-density cell culture, LDH release after H24R4 and/or LPS stimulation was significantly greater than that seen at low-density cell culture (Fig. 4b). Second, the Cx43 inhibitors, heptanol and Gap26, or the GJ enhancer RA, were respectively used to manipulate GJ function composed of Cx43. All of these chemicals had no significant effects on Cx43 protein expression (Fig. 4c), but significantly attenuated or enhanced GJ transmission (Fig. 4d). At low-density cell culture, although the growth of NRK-52E cells was decreased when exposed to H24R4, LPS, or a combination of the two risk factors, pretreatment with heptanol, Gap26, or RA did not affect NRK-52E cell growth. However, when NRK-52E was cultured at high-density cell culture, the roles of GJ inhibitors and enhancers were shown obviously: heptanol or Gap26 reduced cell damage, while RA exacerbated cell damage (Fig. 4e). At high-density cell culture, LDH release was reduced significantly by heptanol or Gap26, while RA significantly increased LDH release. Heptanol, Gap26, or RA, when used at low-density cell culture, had no significant impact on post-hypoxic cellular LDH release either in the presence or absence of LPS stimulation (Fig. 4f). Heptanol, Gap26, and RA themselves or their solvent, DMSO, had no significant effects on the above-mentioned parameters (Supplementary Fig. 4A, B). Finally, to further determine the role of Cx43 in H/R and/or LPS-induced cell damage, specifically, Cx43 was knocked down by special siRNA targeting Cx43, and “parachute” dye-coupling assay showed that Cx43–siRNA decreased the function of GJs significantly in NRK-52E cells (Fig. 5a, b). Although Cx43 knockdown per se did not affect cell growth, it restored H24R4, LPS, or a combination of H24R4 and LPS-induced cell growth depression, and decreased LDH release (Fig. 5c, d). These three methods verified the fact that alternation of Cx43 channel function could regulate H/R and/or LPS-induced renal cell damage.

### Cx43 channels could regulate the content of ROS induced by H24R4 and/or LPS

**Fig. 6:**
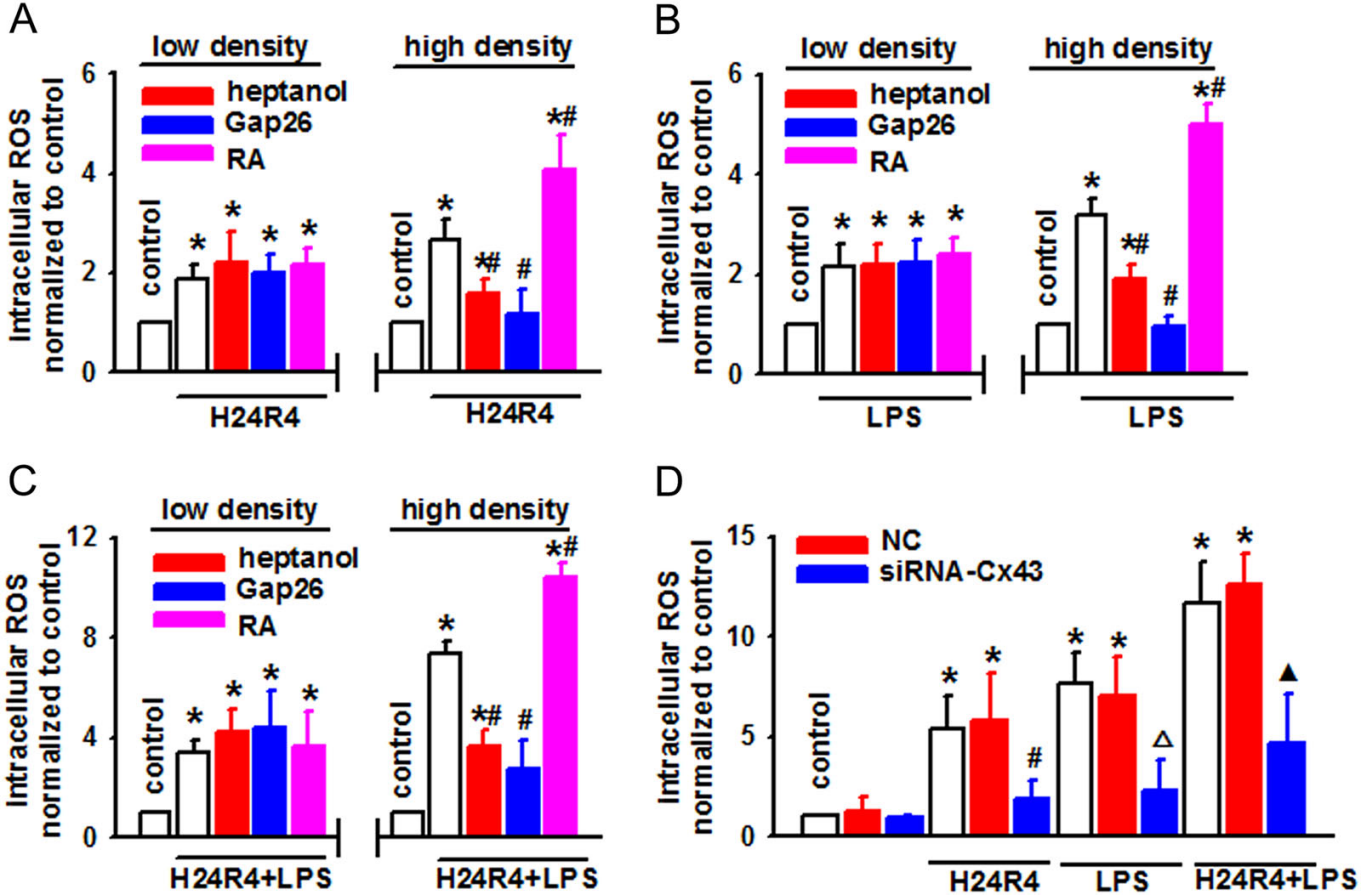
ROS production in NRK-52E cells induced by H24R4 or/and LPS was regulated by Cx43 channels. **a**–**c** Effects of heptanol (2 mM, 1 h), Gap26 (300 μM, 1 h), and RA (10 μM, 24 h) on ROS production at low- and high-density cell culture exposed to H24R4, LPS (5 μg/ml, 24 h), or a combination of H24R4 and LPS. *n* = 6, *P < 0.05 vs control; ^#^*P* < 0.05 vs the same treatment groups at low-density cell culture. **d** Specific siRNA decreased the content of ROS when exposed to H24R4, LPS (5 μg/ml, 24 h), or a combination of H24R4 and LPS. *n* = 5, *P < 0.05 vs control; ^#^*P* < 0.05 vs the H24R4 group; △*P* < 0.05 vs the LPS group; ▲*P* < 0.05 vs H24R4 + LPS group

The results above confirmed that the alteration of Cx43 channel function could affect H/R and/or LPS-induced renal cell damage, the biological basis of which was always relative with the alteration of signal transduction through Cx43 channels. ROS, including oxygen radicals and nonradical compounds, is but one of the signals that could be transmitted through Cx43 channels. Thus, in this section, we investigated the effects of Cx43 channels on the content of ROS. The results showed that at low-density cell culture, heptanol, Gap26, and RA had no effects on ROS production in NRK-52E cells when being subjected to H24R4, LPS, or their combination, while, at high-density cell culture, ROS production was attenuated when Cx43 channel function was inhibited with heptanol and Gap26, but being amplified when Cx43 channel function was enhanced with RA (Fig. 6a–c). Heptanol, Gap26, and RA themselves or their solvent, DMSO, had no significant effects on the above-mentioned parameters (Supplementary Fig. 4C). Moreover, when Cx43 was knocked down by special siRNA, ROS production was most profoundly reduced in NRK-52E cells subjected to H24R4 and/or LPS (Fig. 6d). In order to confirm the effects of Cx43 downregulation on ROS production, we designed another siRNA targeting Cx43 (siRNA–Cx43-1) on NRK-52E cells or human kidney tubular epithelial cells (HK2). Supplementary Fig. 5 showed that siRNA–Cx43-1 could attenuate Cx43 expression on NRK-52E cells effectively (Supplementary Fig. 5A). When Cx43 was knocked down by siRNA–Cx43 and siRNA–Cx43-1, ROS production was reduced on NRK-52E cells and HK2 cells subjected to H24R4 and/or LPS (Supplementary Fig. 5B–D).

### Cx43 GJ inhibition attenuated oxidative stress and inflammatory reaction of NRK-52E cells via mediating the content of ROS

**Fig. 7:**
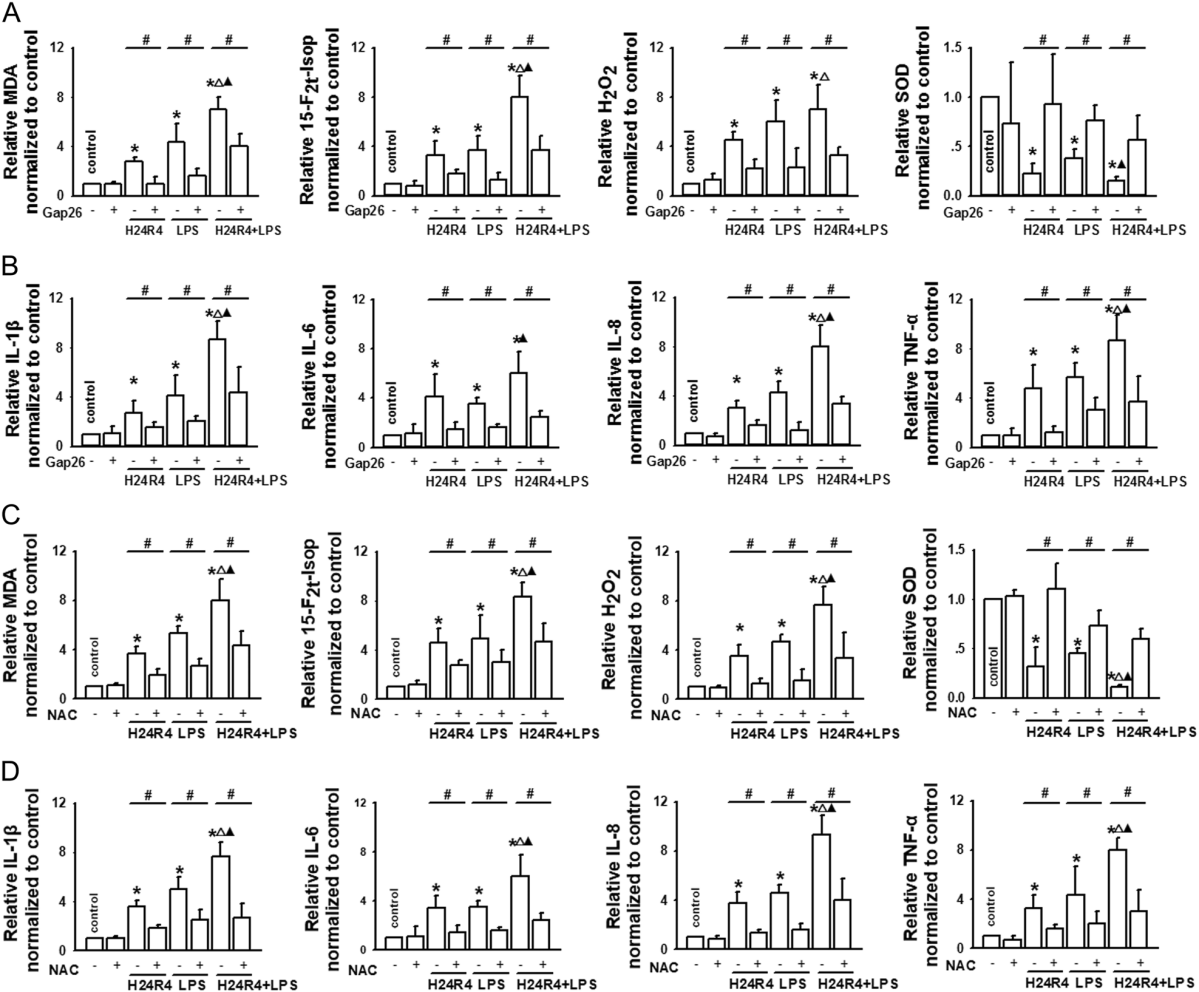
Cx43 channel inhibition attenuated oxidative stress and inflammatory reaction via depressing ROS in vitro. **a** Gap26 application (300 μM, 1 h) attenuated H24R4, LPS, or a combination of H24R4 and LPS-induced 15-F_2t_-Isoprostane (15-F_2t_-Isop), malondialdehyde (MDA), and H_2_O_2_ increase, but increased SOD production. **b** Gap26 application (300 μM, 1 h) attenuated H24R4, LPS, or a combination of H24R4 and LPS-induced IL-1β, IL-6, IL-8, and TNF-α increase. **c** NAC application (10 mM, 1 h) attenuated H24R4, LPS, or a combination of H24R4 and LPS-induced 15-F_2t_-Isop, MDA, and H_2_O_2_ increase, but increased SOD production. **d** NAC application (10 mM, 1 h) attenuated H24R4, LPS, or a combination of H24R4 and LPS-induced IL-1β, IL-6, IL-8, and TNF-α increase. *n* = 5–7, *P < 0.05 vs control; ^#^*P* < 0.05 vs the H24R4 group; △*P* < 0.05 vs the LPS group; ▲*P* < 0.05 vs H24R4 + LPS group. Vehicle control of Gap26 was DMSO, which had no significant effects on the above-mentioned parameters (data not shown)

Figure 6 demonstrated that Cx43 channels could regulate the content of ROS between the neighboring cells, and importantly, ROS was always hypothesized as the “motor” of oxidative stress and inflammation reaction. Therefore, we observed that whether Cx43 channels could affect oxidative stress and inflammation reaction through regulating the content of ROS. As shown in Fig. 7a, when Cx43 channel function was inhibited by Gap26, the levels of 15-F_2t_-Isoprostane (specific index of ROS-induced oxidative stress), the lipid peroxidation product MDA and H_2_O_2_, induced by H24/R4, LPS, or their combination were reduced significantly, accompanied with an increased antioxidant enzyme SOD. Meanwhile, inflammation factors IL-1β, IL-6, IL-8, and TNF-α triggered by H24/R4, LPS, or their combination were also decreased by Gap26 (Fig. 7b). When Cx43 channel function was inhibited by siRNA–Cx43 or siRNA–Cx43-1, the levels of 15-F_2t_-Isoprostane, MDA, and H_2_O_2_ induced by H24/R4, LPS, or their combination were reduced significantly, accompanied with an increased antioxidant enzyme SOD. Meanwhile, inflammation factors IL-1β, IL-6, IL-8, and TNF-α triggered by H24/R4, LPS, or their combination were also decreased (Supplementary Fig. 6).

We used NAC, a kind of ROS scavenger, to eliminate ROS, and found the similar results as Cx43 channel inhibition with Gap26 pretreatment, manifested as the improvement of oxidative stress (MDA, H_2_O_2_, 15-F_2t_-Isoprostane, and SOD) and inflammation reaction (IL-1β, IL-6, IL-8, and TNF-α) caused by H24/R4, LPS, or their combination (Fig. 7c, d).

Combined with the above results that (1) Cx43 channels could regulate the content of ROS (Fig. 6); (2) Cx43 channels could regulate the state of oxidative stress and inflammation reaction caused by H24/R4, LPS, or their combination (Fig. 7a, b); (3) ROS clearance with NAC could improve oxidative stress and inflammatory response caused by H24/R4, LPS, or their combination, we concluded that inhibition of Cx43 channel function could attenuate oxidative stress and inflammatory reaction of NRK-52E cells caused by H24/R4, LPS, or their combination via reducing the content of ROS.

### Cx43 GJ inhibition attenuated oxidative stress and inflammatory reaction of kidneys following AOLT via mediating the content of ROS

**Fig. 8:**
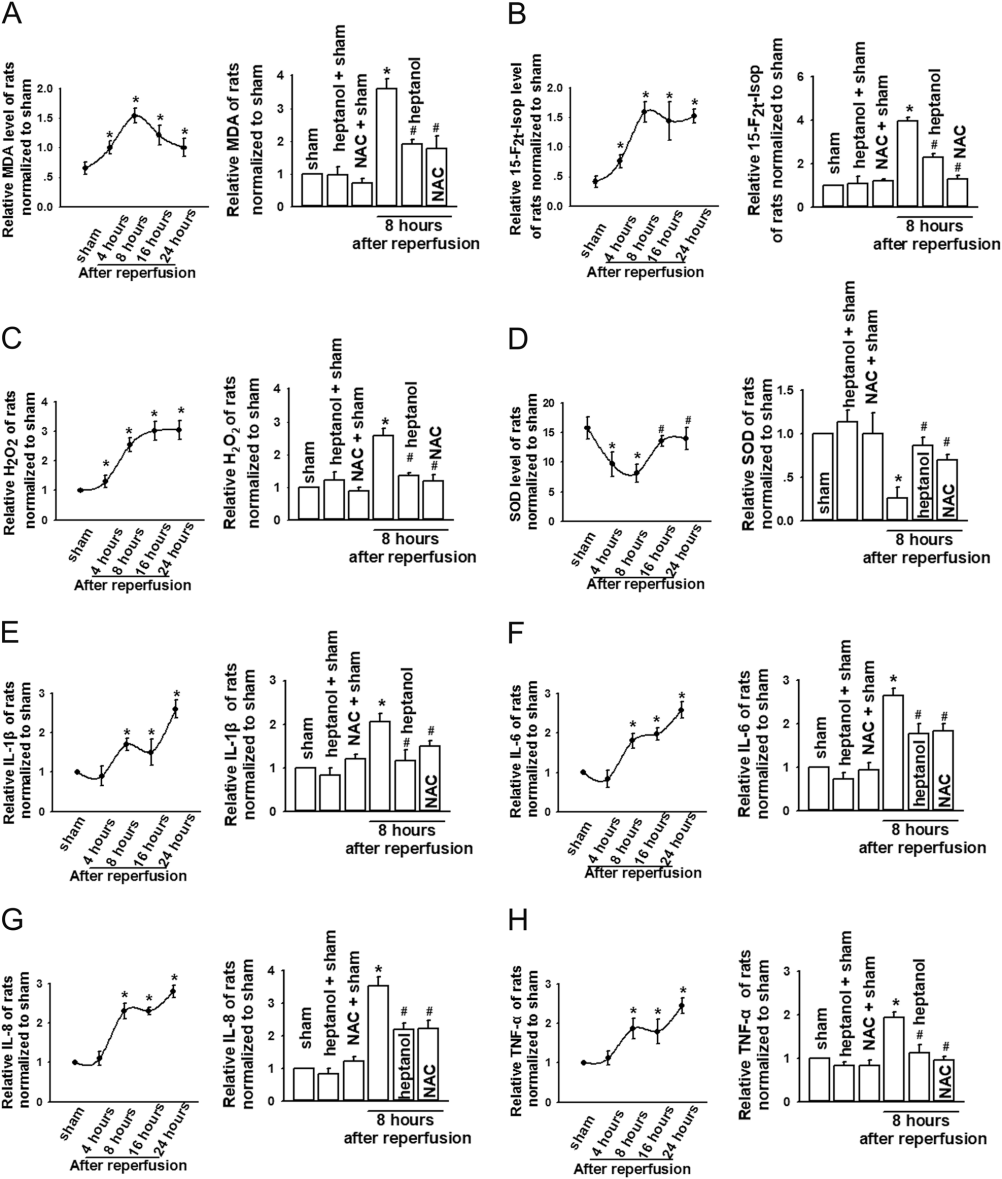
Cx43 channels inhibition attenuated oxidative stress and inflammatory reaction via depressing ROS in vivo. **a**–**d** Changes in 15-F_2t_-Isop, MDA, H_2_O_2_, and SOD at different reperfusion time points after AOLT. Heptanol (0.1 mg/kg, 1 h before AOLT) and NAC (200 mg/kg, 1 h before AOLT) attenuated the level of 15-F_2t_-Isop, MDA, H_2_O_2_, and SOD at 8 h after reperfusion. *n* = 8, *P < 0.05 vs sham; ^#^*P* < 0.05 vs the AOLT group. Vehicle control of heptanol was DMSO, which had no significant effects on the above-mentioned parameters (data not shown). **e–h** Changes in IL-1β, IL-6, IL-8, and TNF-α at different reperfusion time points after AOLT. Heptanol (0.1 mg/kg, 1 h before AOLT) and NAC (200 mg/kg, 1 h before AOLT) attenuated the level of IL-1β, IL-6, IL-8, and TNF-α at 8 h after reperfusion. *n* = 8, *P < 0.05 vs sham; ^#^*P* < 0.05 vs the AOLT group. Vehicle control of heptanol was DMSO, which had no significant effects on the above-mentioned parameters (data not shown)

In order to further confirm the conclusion that alternation of Cx43 channel function could affect the state of oxidative stress and inflammatory reaction of kidneys following AOLT via mediating the content of ROS, we pretreated rat AOLT models with heptanol and NAC. The results showed that the levels of 15-F_2t_-Isoprostane, MDA, and H_2_O_2_ levels reached to a peak 8 h after reperfusion, while SOD declined to the nadir at this moment (Fig. 8a–d). This is consistent with severe functional impairment and pathological damage as shown in Fig. 2d, e. Cx43 channel inhibitor, heptanol, attenuated the productions of 15-F_2t_-Isoprostane, MDA, and H_2_O_2_, and increased the level of SOD at 8 h after reperfusion (Fig. 8a–d). When rats were pretreated with NAC (a kind of ROS scavenger), the results about the changes in oxidative stress and inflammatory reaction of kidneys following AOLT were similar with that of rats pretreated with heptanol (Fig. 8a–d). Inflammation factors IL-1β, IL-6, IL-8, and TNF-α, also increased rapidly during the first 8 h after reperfusion and kept at the same levels until 16 h, and then continued to rise as time extended (Fig. 8e–h), which were all reduced by heptanol and NAC (Fig. 8e–h). DMSO had no significant effects on the above-mentioned parameters (data not shown).

Of note, the alteration patterns of oxidative stress and inflammatory reaction were different: 15-F_2t_-Isoprostane, MDA, and H_2_O_2_ levels all reached to the peak, and SOD declined to the nadir at 8 h after reperfusion, but IL-1β, IL-6, IL-8, and TNF-α all continued to increase after that moment (Fig. 8).

### Cx43 GJ inhibition attenuated RIP1 and MLKL expression in vivo and in vitro via mediating the content of ROS

**Fig. 9:**
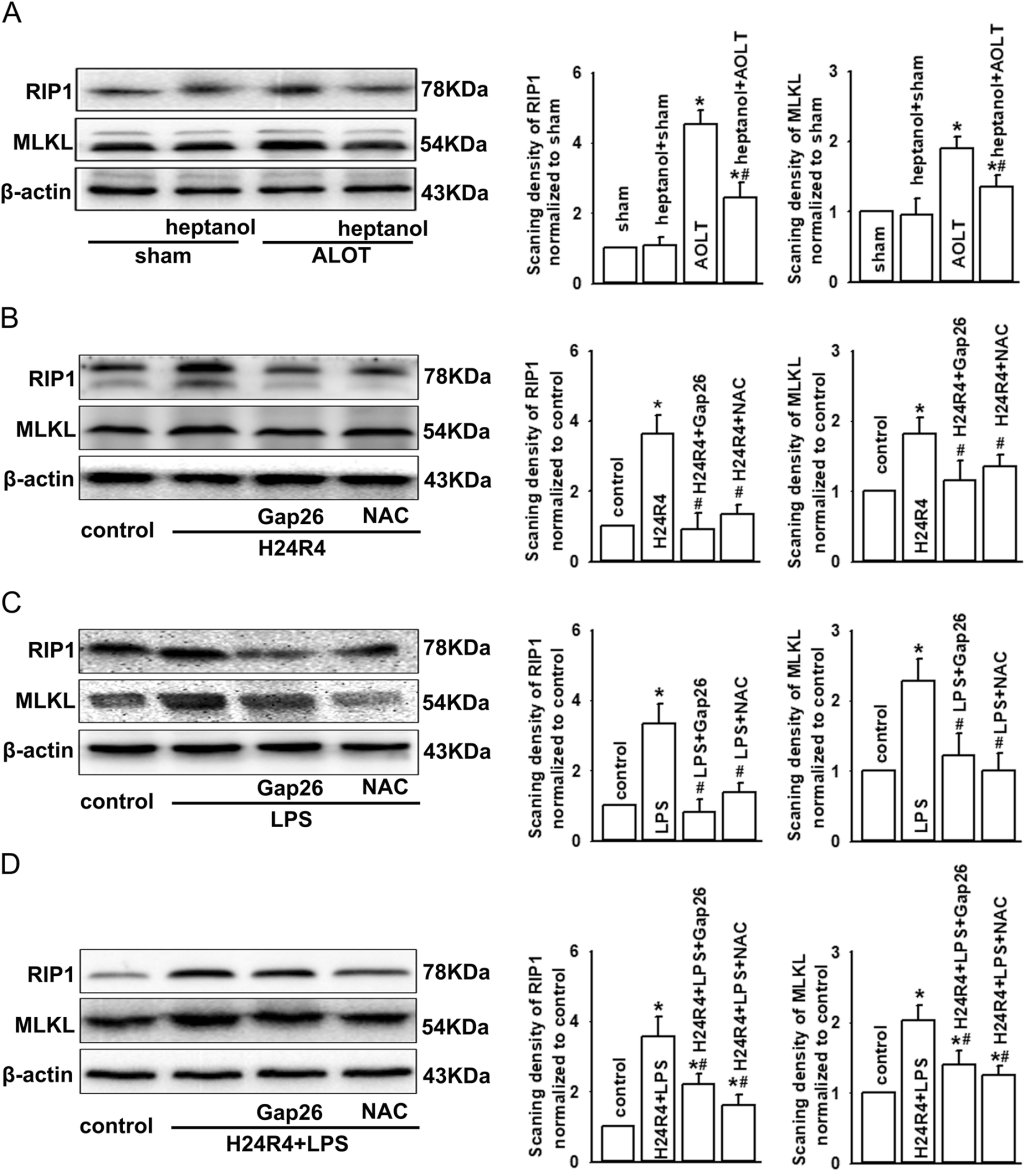
Cx43 GJ inhibition attenuated RIP1 and MLKL expression in vivo and in vitro via mediating the content of ROS. **a** Heptanol (0.1 mg/kg, 1 h before AOLT) attenuated RIP1 and MLKL expression at 8 h after reperfusion. *n* = 8, *P < 0.05 vs sham; ^#^*P* < 0.05 vs the AOLT group. Vehicle control of heptanol was DMSO, which had no significant effects on the above-mentioned parameters (data not shown). **b** Gap26 application (300 μM, 1 h) or NAC application (10 mM, 1 h) attenuated RIP1 and MLKL expression when NRK-52E cells were pretreated with H24R4. *n* = 8, *P < 0.05 vs control; ^#^*P* < 0.05 vs the H24R4 group. **c** Gap26 application (300 μM, 1 h) or NAC application (10 mM, 1 h) attenuated RIP1 and MLKL expression when NRK-52E cells were pretreated with LPS. *n* = 8, *P < 0.05 vs control; ^#^*P* < 0.05 vs the LPS group. **d** Gap26 application (300 μM, 1 h) or NAC application (10 mM, 1 h) attenuated RIP1 and MLKL expression when NRK-52E cells were pretreated with H24R4 + LPS. *n* = 8, *P < 0.05 vs control; ^#^*P* < 0.05 vs H24R4 + LPS group

We have found that oxidative stress and inflammatory response played an important role in this process of AKI induced by LT (Figs. 7, 8), which might result in necroptosis^16,17^, which prompts us to consider whether AKI induced by LT is relative with necroptosis. Thus, we explored the effects of Cx43 transmitting ROS on RIP1 and MLKL expression, both of which are considered to be the typical markers of necroptosis. Figure 9A demonstrated that both RIP1 and MLKL expression were increased obviously after AOLT. When the rats were pretreated with Cx43 channels inhibitor, heptanol, RIP1, and MLKL expression were attenuated. The results obtained in vitro also illustrated the same variation tendency: when NRK-52E cells were pretreated with H24R4, LPS, or both, RIP1 and MLKL expression increased significantly, which could be reversed by Cx43 channels specific inhibitor, Gap26 or ROS scavenger, NAC (the changes in pMLKL were also consistent with those of MLKL, Supplementary Fig. 7). Necrostain-1 (NEC-1) was used to inhibit necroptosis; NEK-52E cells damage was attenuated significantly (Supplementary Fig. 7). Therefore, we concluded that Cx43 transmitting ROS could regulate expression of RIP1 and MLKL on kidneys, ultimately resulting in necroptosis.

## Discussion

In this study, we found that the influence of postoperative AKI on patients’ recovery and survival rate was striking. Given that renal hypoperfusion and endotoxin renal toxicity are two independent risk factors of AKI following LT^4,24^, and that Cx43 overexpression exacerbates renal cell injury^25,26^, we determined the influence of Cx43 on post-hypoxic and post-AOLT renal injuries and complications. The data showed that Cx43 inhibition attenuated AKI following LT in vivo and decreased H/R, LPS, or the combination of H/R and LPS-induced NRK-52E cell damage in vitro. Cx43 channel function alternation regulated the content of ROS between the neighboring cells, which not only resulted in the changes in oxidative stress and inflammatory reaction, but also altered the expression of RIP1 and MLKL, the typical markers of necroptosis, ultimately influencing renal injuries. These findings indicated that Cx43 played a vital role in AKI following LT.

Our results showed that the level of Cx43 expression on donor liver tissue was consistent with liver pathological damage, which prompts us that alternation of Cx43 expression might play an important role in organ damage following LT. However, the acquisition of kidney tissue from patients undergoing LT was almost impossible. With the assistance of kidney transplant surgery colleagues, we obtained six samples of kidney tissues during the operation of kidney transplantation. The results showed that, after reperfusion, both kidney damage and Cx43 expression have no statistical differences (Supplementary Fig. 8). We analyzed the possible reasons and found that maybe the reperfusion time was not enough. Under normal conditions, the kidney transplantation will be finished within 2 h after reperfusion. It means that samples experienced 2 h or less reperfusion time. However, our present results (Fig. 2) prompt us that kidney damage and Cx43 expression were changed at least 4 h after reperfusion. As far as we know, the operation that kidney undergoes I/R injury, especially the reperfusion time exceeding 4 h, is very limited. It is very hard for us to get the proper kidney samples experiencing 4 or more hours of reperfusion. Therefore, in this study, we had to employ rat AOLT models to directly observe the influence of Cx43 on organ damage following LT, especially for the renal damage. Rat AOLT models not only mimicked most procedures of LT, such as SVC, IVC and PV blockade, cold liver protection fluid perfusion, liver I/R, and intestine passive congestion, but also avoided the effects of AOLT-mediated complex complication and rejection^4,27^. Our results from in vivo studies indicated that Cx43 participated in the liver and kidney damages following AOLT, especially when donor livers and kidneys suffered prolonged hypotension and severe endotoxemia.

Our clinical observations show that patients with AKI suffered from significantly longer time with a respirator and staying in the intensive care unit, and had significantly lower survival rate after 30 days, and 1–3 years than patients without AKI. This indicated that AKI had a great impact on the prognosis of patients suffering from LT. Because of the characteristics of the LT operation, such as inferior vena cava occlusion, intestinal ischemia-induced sepsis, and so on, AKI became a large probability event. If AKI continued to deteriorate and amplify, it would definitely affect the outcome of LT patients. Therefore, appropriate interventions to prevent the sustained deterioration and magnification of AKI injury would have a positive impact on the prognosis of LT patients.

GJs mediate direct intercellular movement of cytoplasmic signaling molecules in different organs, which is always considered to be the potent biological basis of organ damage deterioration and magnification, also called the “bystander effect”^28,29^. In this research field, Cx43 was widely explored^30^. In different cases, Cx43 exerted different effects on organ damage^31,32^, the biological basis of which was often related to the different types of signal transmission mediated by Cx43 channels, such as “death signals” and “protection signals”^10^. When the balance between the two different kinds of cell signals was broken, it would exert opposite effects. In our experiments, Cx43 inhibition was beneficial for renal protection, which suggested that “death signals” transfer was predominant in this process. “Death signals” not only damage the neighboring cells directly, but also activate other signal pathways, resulting in cytotoxicity enhancement indirectly^33,34^. During the process of LT, huge surgical trauma resulted in the generation of massive “death signals” and the increase of Cx43 expression in kidneys. “Death signals” continuously transmitted to the neighboring cells via GJs composed of C43, leading to the scope of injury expanded and deteriorated. Therefore, blocking the diffusion of “death signals” to the neighboring cells mediated by GJs composed of Cx43 could effectively limit the damage to a certain extent and prevent the damage from deteriorating and amplifying continuously. That might be a new and effective strategy to protect against AKI following LT.

Although “death signals” had been explored for many years, their intrinsic quality had yet to be identified. ROS was but one of the signals that could be transmitted through Cx43 channels, and always hypothesized as the “motor” of oxidative stress and inflammation reaction. Thus, the possibility of ROS as a “death signal” was very great^4^. Furthermore, we demonstrated that ROS transfer could also be regulated by Cx43 channels. Given that the distribution of Cx43 was much more universal, and its channel permeability was much larger than other connexin channels, we believed that ROS transfer mediated by Cx43 channels would be much more effective in organ or tissue damage than that mediated by other connexin channels, such as Cx32. In order to exclude the influence of Cx32 GJs on our current results, we determined the interaction between Cx32 and Cx43 in NRK-52E cells. Supplementary Fig. 9 showed that Cx32 knockdown with siRNA–Cx32 had no effect on Cx43 expression, while Cx43 knockdown with siRNA–Cx43 also had no effects on Cx32 expression, which indicated that Cx43 and Cx32 functioned individually and did not interact with each other.

ROS was always hypothesized as the “motor” of oxidative stress and inflammation reaction, contributing to organ injuries during LT^35–37^. We have demonstrated that (1) Cx43 channels regulated the content of ROS; (2) ROS clearance with NAC attenuated oxidative stress and inflammation reaction; (3) Cx43 channels inhibition also depressed oxidative stress and inflammation reaction. Thus, we concluded that Cx43 channels influenced the state of oxidative stress and inflammation reaction via regulating the content of ROS, which might be the underlying mechanism of Cx43 inhibition protecting against AKI following LT. Interestingly, the dynamic changes in oxidative stress and inflammation reaction after reperfusion were different: oxidative stress parameters 15-F_2t_-Isoprostane, MDA, and H_2_O_2_ levels reached the peak at 8 h after reperfusion, but inflammatory IL-1β, IL-6, IL-8, and TNF-α continued to increase as time extended. We speculated that they functioned at different stages of AKI following LT. Oxidative stress might react at an earlier stage. This might offer time-specific intervention clinically.

LT is a huge trauma for patients. Its damage to other organs is often acute and severe. Therefore, previous reports^15^, including our present study, all demonstrated that renal pathological injuries induced by LT were necrosis. As reported, severe both oxidative stress and inflammation reaction are the two important factors of necroptosis^16,17^. Thus, we explored the effects of Cx43 transmitting ROS on RIP1 and MLKL expression, the typical markers of necroptosis. The results in Fig. 9 showed that Cx43 transmitting ROS could regulate expression of RIP1 and MLKL in kidneys. All of our findings suggested that inhibiting GJs composed of Cx43 attenuated the content of ROS, which not only blocked the development of oxidative stress and inflammatory reaction, but also attenuated necroptosis. Eventually, renal damage deterioration and magnification were well controlled. Thus, GJs composed of Cx43 might be an effectively therapeutic target for the protection against AKI following LT.

Apart from forming GJs, Cx43 also forms unopposed hemichannels, which provide a pathway for molecular exchange between the cytoplasm and the extracellular compartment^38^. Under the normal condition, hemichannels present a low open probability that could be regulated by a variety of factors, including oxidative stress and inflammatory reaction^39,40^. The opening of hemichannels allows the release of small molecules, providing a paracrine route for intercellular communication^41^. It seems hard to distinguish the functions of hemichannels and GJs in our research. However, modulation of hemichannels and GJ composed of Cx43 was totally different sometimes. For example, it was reported that TNF-α and IL-β depressed the function of GJs, but increased hemichannel activity^42–44^. In our research, we used different cell-density culture to investigate GJ function in NRK-52E cells subjected to H24R4 and/or LPS-induced damage. At low-density cell culture, no GJ was formed, but hemichannels could exist^44^. Gap26 could inhibit hemichannels composed of Cx43^45^, but had no effects on cell growth and LDH release (Fig. 4e, f). These results supported that the effects of Cx43 on H24R4 or LPS-induced NRK-52E cell damage were mediated by GJs, but not hemichannels.

## Materials and methods

### Patients

This study was approved by the Research Ethics Board of the Third Affiliated Hospital, Sun Yat-sen University, China (clinical trial registration number. ChiCTR-OCH-12002255). Informed consent was obtained from each patient. Eighty-two patients (36–60 years old), receiving the first-time orthotopic LT, were investigated. Procedures and managements of anesthesia or operation were performed as routine in our hospital. It is impractical to get kidney tissue of patients undergoing LT, and donor liver experienced the same pathophysiological process as kidneys. Thus, tissues of donor liver from eight patients were obtained before or after reperfusion during the trimming phase to observe LT-induced organ damage and the alternations of Cx43 expression. Normal liver tissues from eight patients with hepatic hemangioma were used as control. Hemodynamics were determined by a Hewlett-Packard M1166A component monitoring system (Hewlett-Packard, Palo Alto, CA, USA). Patients were excluded if they were nephrosis, unable to provide consent, and received dialysis before or during LT. The Model for End-stage Liver Disease (MELD) scores of patients were all over 16. All study enrollment procedures and subsequent data collection and acquisition were approved by the Research Ethics Board.

### Establishment of the rat AOLT model and treatment

Male SD rats, aged 8–10 weeks and weighing 220–250 g, were obtained from Medical Experimental Animal Center of Guangdong Province, China. All study protocols were approved by the Institutional Animal Care and Use Committee of Sun Yat-sen University, Guangzhou, China. Rat AOLT model was described in our previous study^4,46^. In brief, an open-face guard was used to administer the inhalational ether anesthetic until the rats had no response to a needle stimulus. The liver falciform ligament was resected and ligated. Then, the blood vessel was severed along the esophagus. We revealed the liver until the supra hepatic vena cava (SVC) was liberated and placed it back into its original position. A bold line was prepared to use as guideline for SVC blockage. After the upper region of the left renal vein was liberated, we dissociated the inferior vena cava (IVC) and dissected the first hepatic portal and separated the portal vein (PV). Both the hepatic artery and biliary were also liberated successively according to their anatomic relationship, and then, the portal hepatics were ligated. Microvascular clamps were used at the convergence of the inferior mesenteric, splenic veins, hepatic artery, SVC, and IVC. We punctured the PV with a 24-gauge needle in preparation for reperfusion and made one 1-mm incision on the IVC wall as an outflow tract. During reperfusion, pre-cold 4 °C Ringer lactate solution was injected into that outflow tract at 2.5 ml/min until the color of the liver turned to yellow. Then, the needle was extracted, and the openings of the PV and IVC were closed by using 8–0 sutures. Finally, the PV, SVC, IVC, and hepatic artery were unclamped. The anhepatic phase lasted for about 20 ± 1 min. During operation, the amount of bleeding was <1.5 ml, and 3 ml of Ringer lactate solution was continuously pumped into the caudal vein. Compound lidocaine cream (Ziguang Pharmaceutical Co., Ltd., Beijing, China) was smeared onto the cut surface. After operation, all rats were maintained in a temperature-controlled environment with a 12-h light–dark cycle with free access to water^15^.

According to the corresponding experimental groups, rats were respectively intraperitoneally pretreated with heptanol at 0.1 mg/kg (Sigma-Aldrich) or NAC (Sigma-Aldrich) at 200 mg/kg for 1 h before AOLT.

### Mean arterial pressure (MAP) assay

The right femoral arteries were catheterized with a polyethylene catheter (outer diameter, 0.965 mm; inner diameter, 0.58 mm) for monitoring mean arterial pressure^15^, which was recorded before this operation, and when PV, SVC, IVC, and hepatic artery were clamped. Also, mean arterial pressure was recorded at different time points (1–30 min), when PV, SVC, IVC, and hepatic artery were unclamped.

### Assessment of tissue specimens

Kidney specimens from rats and liver specimens from patients were fixed in 10% buffered formalin, embedded in paraffin, and processed for hematoxylin–eosin staining. Immunohistochemical staining was performed in 4-μm paraffinized sections for Cx43 protein detection as described^15^. After being dewaxed and dehydrated, the sections were incubated with H_2_O_2_ (3%) to inhibit endogenous peroxidase activity. The slides were incubated with primary antibodies against Cx43 (Sigma-Aldrich, 1:1000) over night at 4 °C. After incubation with its corresponding secondary antibody (Sigma-Aldrich, 1:4000), the samples were visualized with a light microscope (EclipseE800, Nikon, Tokyo, Japan).

### “Scrape and load” assay

Rats were treated with heptanol (0.1 mg/kg) or its solvent DMSO intraperitoneally 1 h before AOLT. The dose in rats was chosen based on literature^22^ that heptanol at 0.1 mg/kg could effectively inhibit Cx43, and our preliminary study showed that it did not induce liver injury or change Cx43 expression in livers or kidneys at this dose (Supplementary Fig. 3). One hour later, kidneys were excised and freshly sliced and processed as described^47^. We used a 27-gauge needle dipping into a solution containing 0.5% Lucifer Yellow (Invitrogen) and 0.5% Rhodamine (Invitrogen). Lucifer Yellow could be transferred through GJs, and in contrast Rhodamine is impermeable. The needle was used to mechanically penetrate a small area of each slice to apply dyes. After 5 min of incubation, kidney slices were rinsed in saline, fixed in 4% paraformaldehyde for 30 min, frozen in OCT compound, and cyro-sectioned into 10-µm sections. The slices were then rinsed in saline again, and mounted and imaged by fluorescence microscopy. Quantitative analysis of the distance of dye spread was performed between the front of dye transfer and the scrape line.

### Cell culture and treatments

NRK-52E cells, a cell line of rat kidney tubular epithelial cell origin, were obtained from American Type Culture Collection (Manassas, VA, USA) and cultured in DMEM/F-12 supplemented with 10% fetal bovine serum. Cells were grown at 37 °C in an atmosphere of 5% CO_2_ in air^48^, and then pretreated with connexin channel inhibitors heptanol, 2 mM, for 1 h (Sigma-Aldrich, a Cx43 uncoupler)^49^ or Gap26, 300 μM, for 1 h (Sigma-Aldrich, a connexin mimetic peptide)^50^; a Cx43 expression enhancer, retinoic acid (RA), 10 μM, for 24 h (Sigma-Aldrich)^4^; NAC (Sigma-Aldrich, a kind of ROS scavenger), 10 mM, for 1 h before H/R or/and LPS exposure. “Parachute” dye-coupling assay was performed as described below. The solvents of heptanol and RA were DMSO.

### NRK-52E cells exposed to H/R or LPS

Renal hypoperfusion induced by hypotension is significant during LT, which results in renal I/R injury. Thus, we used NRK-52E cell H/R model to mimic renal cell I/R injury. NRK-52E cells were cultured in low-oxygen condition (95% N_2_ + 5% CO_2_) for 24 h in a humidified hypoxia chamber (Galaxy 48 R; Eppendorf, Hamburg, Germany) before being re-oxygenated by exposing to normal-oxygen condition (95% air + 5% CO_2_) for 4 h (H24R4). Cells in the control groups were cultured in normoxic conditions for 28 h without exposing to H/R^4^. NRK-52E cells in subgroups were pretreated with LPS (Sigma-Aldrich, 5 μg/ml) for 28 h in the absence or presence of H/R stimulation, and were then harvested for assays as described below^51^.

### Assays for Cell Counting Kit-8 (CCK8), lactate dehydrogenase (LDH), and intracellular reactive oxygen species (ROS), and assessments of creatinine (Cr) and blood urea nitrogen (BUN), and intracellular 15-F_2t_-Isoprostane, malondialdehyde (MDA), H_2_O_2_, SOD, IL-1β, IL-6, IL-8, TNF-α, and LPS

NRK-52E cells were seeded at low density (25000 cells/cm^2^, no GJIC formed), or high density (125,000 cells/cm^2^, GJIC formed) in 24-well plates. At the end of different stimulations, CCK8 and LDH assays were carried out according to the manufacturer’s instruction (Dojindo, Tokyo, Japan). Intracellular ROS production was estimated by using 2,7-dichlorofluorescein diacetate (Sigma-Aldrich)^4^. Cr and BUN were measured in blood samples with an automatic biochemistry analyzer (Hitachi 7600-020/7170 A, Tokyo, Japan). Kidney specimens were fixed in 10% buffered formalin, embedded in paraffin, and processed for H&E staining. 15-F_2t_-Isoprostane, MDA, H_2_O_2_, SOD, IL-1β, IL-6, IL-8, and TNF-α are determined by the respective assay kits following the manufacturers’ instructions (Sigma-Aldrich), and the samples used for measurement were from cells in vitro and rat renal tissues in vivo. LPS was determined by its assay kits following the manufacturers’ instructions (USBiological, Swampscott, MA, USA), and the blood samples used for measurement were from patients or rats.

### “Parachute” dye-coupling assay

Functional GJIC was examined with “Parachute” dye-coupling assay as described^52^.

### Inhibition of Cx43 expression by small-interfering RNA (siRNA) transfection

Cells were transfected with specific siRNA (siRNA–Cx43 shown in the figures: GCTGGTTACTGGTGACAGA. SiRNA–Cx43-1: CCGCAATTACAACAAGCAA shown in the supplemental figures) targeting Cx43 gene or a nonspecific control siRNA (NC as shown in the figures). Transfection into NRK-52E cells was carried out by using Lipofectamine 2000 (Invitrogen, Carlsbad, CA, USA) according to the manufacturer’s instructions^53^.

### Western blotting

Western blotting followed the standard procedures as described^53^. Anti-Cx43 (Sigma-Aldrich, 1:4000) and its corresponding secondary antibody (1:5000) were used to detect Cx43 expression, while anti-β-Tubulin and its corresponding secondary antibodies were used at 1:4000. Anti-RIP1 (Sigma-Aldrich, 1:2000) and anti-MLKL (Sigma-Aldrich, 1:2000) were used to detect RIP1 and MLKL expression, while anti-β-actin and its corresponding secondary antibodies were used at 1:4000.

### Statistical analysis

Statistical analysis was performed with SPSS 15.0 software (SPSS Inc., Chicago, IL). In Table 1, time of respirator use, intensive care unit residence time, and hospital time are presented as median with interquartile range. Patient survival is presented as rates. The time comparison was made by U Mann–Whitney rank-sum test, and the rate comparison was made by chi-square test or Fisher’s exact probability method. All curve fitting was made by Sigmaplot 10.0 (Systat Software, Inc., Chicago, IL) and formed by the graph properties, by using the function of smoothed (spline) in the shape property. For data obtained from in vivo and in vitro experiments, multiple comparisons were analyzed with repeated measures one-way ANOVAs followed by Tukey post hoc comparisons.

## Supplementary information

supplemental materimals
