# Supplementary material for: Inhibition of gap junction composed of Cx43 prevents against acute kidney injury following liver transplantation
Source: Cell Death Dis. 2019 Oct 10;10(10):767. doi: 10.1038/s41419-019-1998-y (PMC6787008; doi:10.1038/s41419-019-1998-y)
Supplement: Supplementary file 1 — supplemental materimals [file 41419_2019_1998_MOESM1_ESM.pdf]

Supplemental Data

Supplemental Figure 1

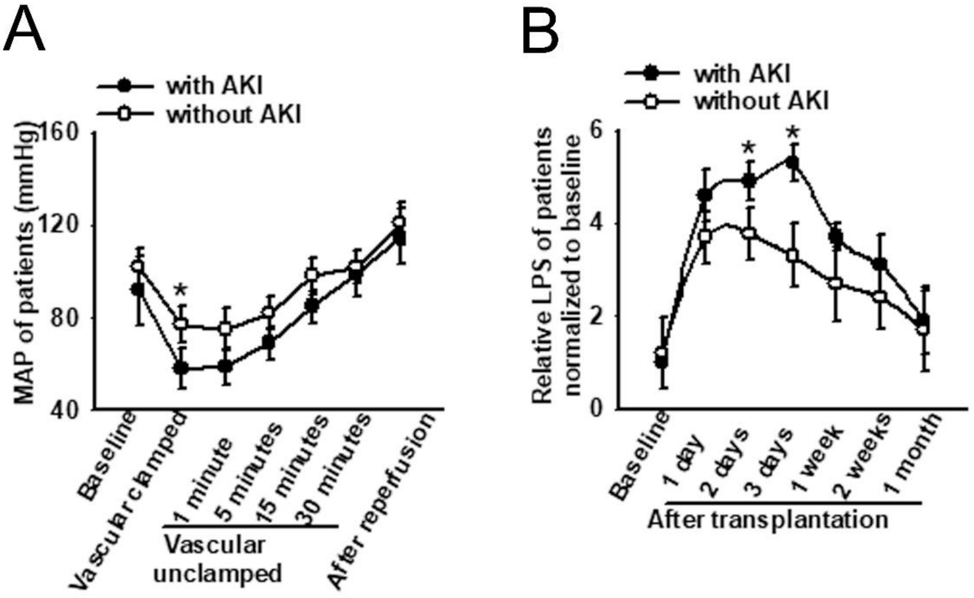

Supplemental Figure 1. Changes of patients' MAP and LPS during LT.

(A) Changes of patients' MAP during LT. (B) Changes of patients' LPS at different reperfusion time points after LT. Among these 82 patients, 38 patients are with AKI after LT, while 44 patients are without AKI after LT. *P < 0.05 vs the same point.

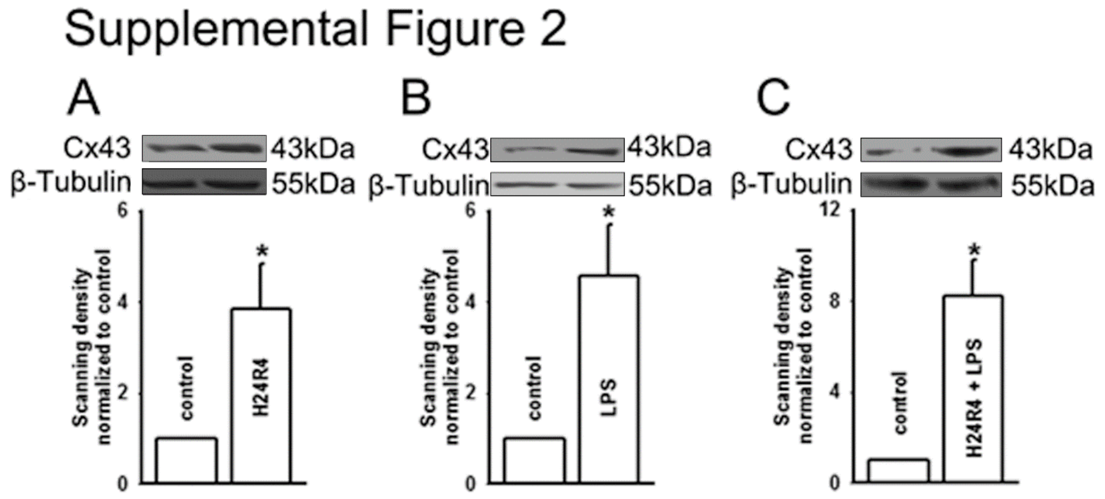

Supplemental Figure 2. Cx43 expression on NRK-52E cells when exposed to H24R4, LPS or combination of H24R4 and LPS.

(A) Cx43 expression on NRK-52E cells when exposed to H24R4. (B) Cx43 expression on NRK-52E cells when exposed to LPS (5 μg/ml, 24 hours). (C) Cx43 expression on NRK-52E cells when exposed to the combination of H24R4 and LPS (5 μg/ml, 24 hours). n = 6, $^{*}P < 0.05$ *vs* control.

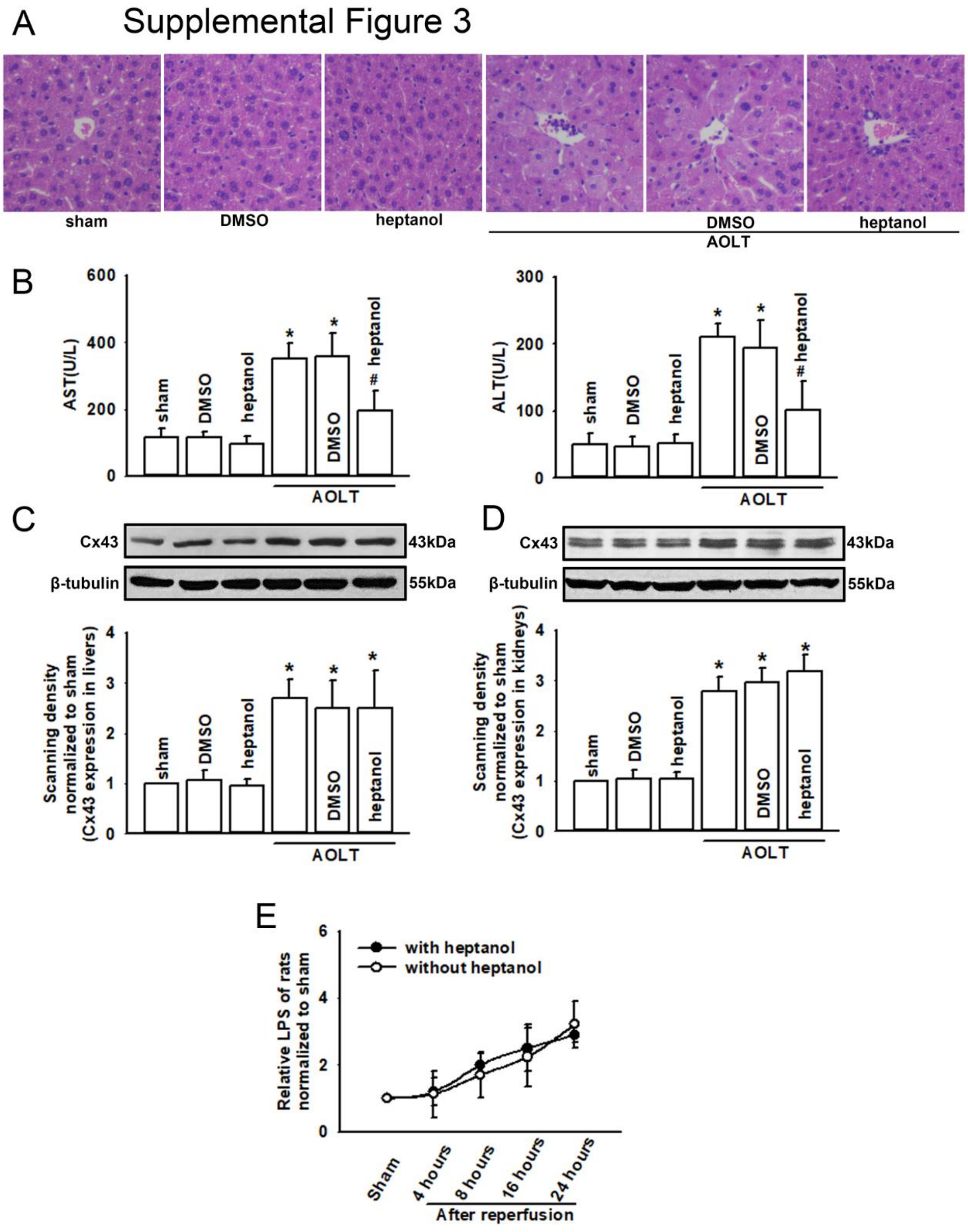

Supplemental Figure 3. Effects of heptanol on rat liver damage, Cx43 expression in rat livers or kidneys, or the level of LPS during AOLT of rats.

(A) Heptanol (0.1 mg/kg) itself had no effects on liver damage of rats, but it attenuated liver damage induced by AOLT (H&E staining; original magnification ×200), n = 8. (B) Heptanol (0.1 mg/kg) itself had no effects on the levels of ALT and

AST, but it attenuated liver function impairment induced by AOLT. n = 8. (C-D) Heptanol (0.1 mg/kg) itself had no effects on Cx43 expression in rat livers or kidneys, n = 3. (E) Heptanol (0.1 mg/kg) itself had no effects on the level of LPS during AOLT of rats, n = 5.

Supplement Figure 4

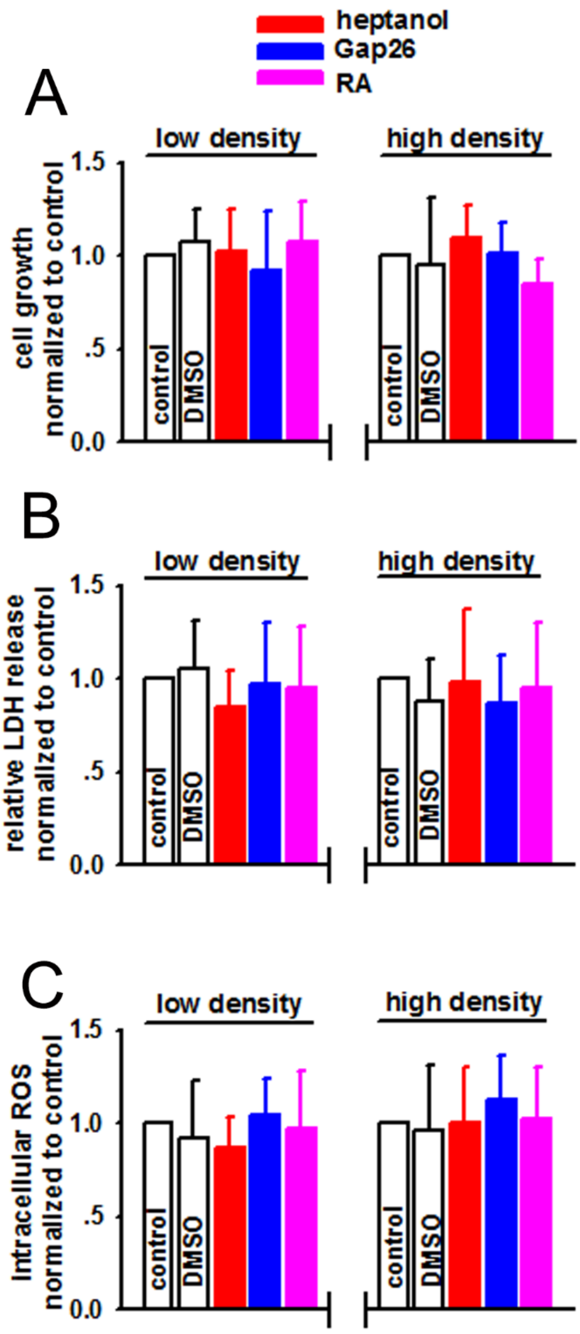

Supplemental Figure 4. Effects of DMSO, heptanol, Gap26 and RA on NRK-52E cells at low and high-density cell culture.

(A) Effects of DMSO, heptanol (2mM, 1 hour), Gap26 (300μM, 1 hours) and RA (10 μM, 24 hours) on cell growth at low and high-density cell culture. (B) Effects of DMSO, heptanol (2 mM, 1 hour), Gap26 (300μM, 1 hours) and RA (10 μM, 24 hours)

on LDH release at low and high-density cell culture. (C) Effects of DMSO, heptanol (2 mM, 1 hour), Gap26 (300 μM, 1 hours) and RA (10 μM, 24 hours) on ROS production at low and high-density cell culture. n = 5.

Supplemental Figure 5

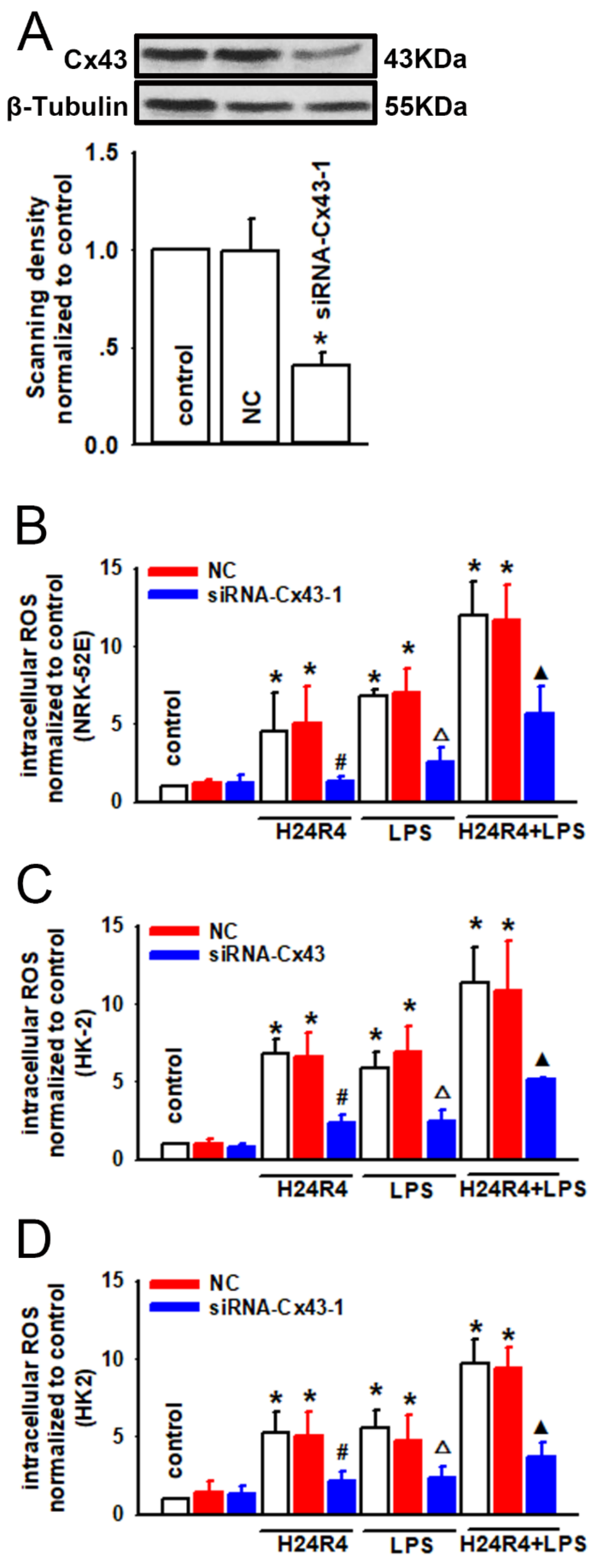

Supplemental Figure 5. ROS production in NRK-52E cells and HK2 cells

induced by H24R4 or/and LPS was attenuated by siRNAs targeting Cx43.

(A) Cx43 expression in NRK-52E cells was down-regulated by siRNA-Cx43-1 (which is different from the prior siRNA-Cx43). n = 5, *P < 0.05 vs control. (B-D) New siRNA-Cx43-1 decreased the content of ROS when exposed to H24R4, LPS (5 μg/ml, 24 hours) or combination of H24R4 and LPS. n = 5, *P < 0.05 vs control; #P < 0.05 vs H24R4 group; △P < 0.05 vs LPS group; ▲P < 0.05 vs H24R4+LPS group.

Supplemental Figure 6

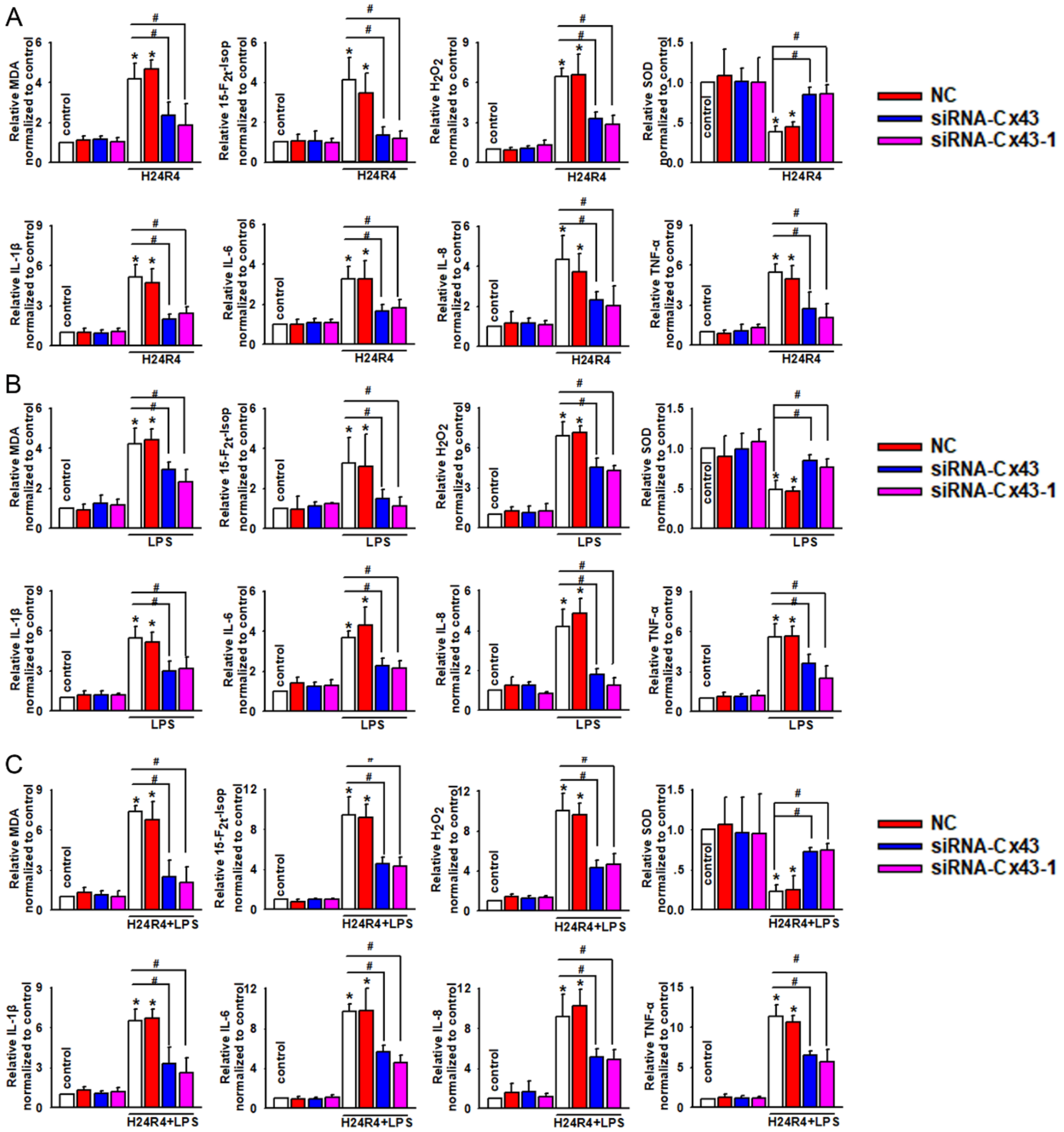

Supplemental Figure 6. Cx43 channels inhibition attenuated oxidative stress and inflammatory reaction in vitro.

(A) New siRNA-Cx43-1 attenuated H24R4 induced 15-F2t-Isop, MDA, H_2O_2, IL-1β, IL-6, IL-8 and TNF-α increase, but increased SOD production. (B) New siRNA-Cx43-1 attenuated LPS induced 15-F2t-Isop, MDA, H_2O_2, IL-1β, IL-6, IL-8 and TNF-α increase, but increased SOD production. (C) New siRNA-Cx43-1 attenuated H24R4+LPS induced 15-F2t-Isop, MDA, H_2O_2, IL-1β, IL-6, IL-8 and TNF-α increase, but increased SOD production. n = 5, *$P < 0.05$ vs control, #$P < 0.05$.

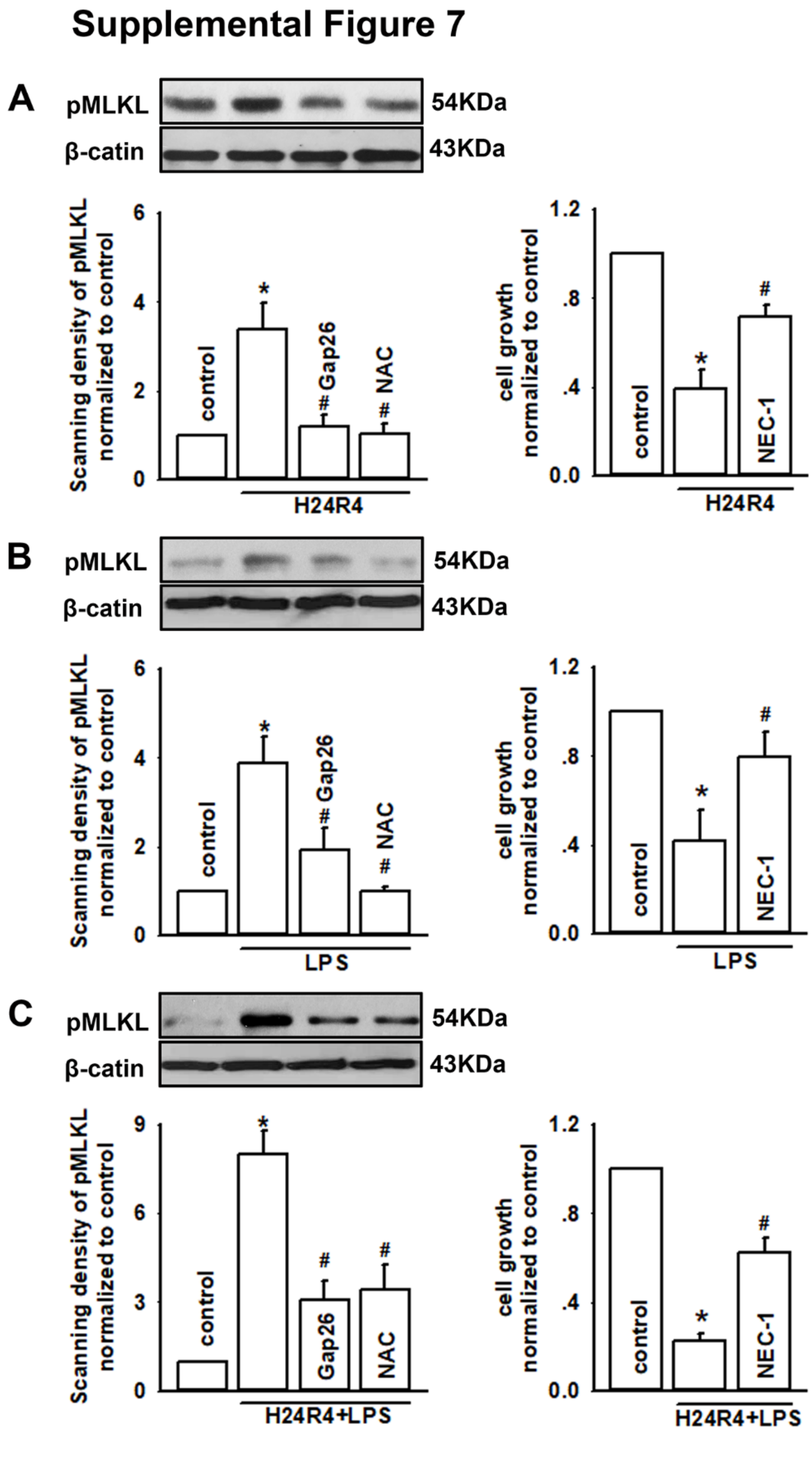

Supplemental Figure 7. Cx43 GJ inhibition attenuated pMLKL expression in vitro via mediating the content of ROS. The inhibitor of necroptosis, necrostain-1 (NEC-1) attenuated cell damage induced by H24R4, LPS or H24R4+LPS.

(A) Gap26 application (300 μM, 1 hour) or NAC application (10 mM, 1 hour) attenuated pMLKL when NRK-52E cells were pretreated with H24R4. n = 4, *P < 0.05 vs control; #P < 0.05 vs H24R4 group. NEC-1 application (30 μM, 24 hours) attenuated cell damage induced by H24R4. n = 4, *P < 0.05 vs control; #P < 0.05 vs H24R4 group. (B) Gap26 application (300 μM, 1 hour) or NAC application (10 mM, 1 hour) attenuated pMLKL expression when NRK-52E cells were pretreated with LPS. n = 8, *P < 0.05 vs control; #P < 0.05 vs LPS group. NEC-1 application (30 μM, 24 hours) attenuated cell damage induced by LPS. n = 4, *P < 0.05 vs control; #P < 0.05 vs LPS group. (C) Gap26 application (300 μM, 1 hour) or NAC application (10 mM, 1 hour) attenuated pMLKL expression when NRK-52E cells were pretreated with H24R4+LPS. n = 8, *P < 0.05 vs control; #P < 0.05 vs H24R4+LPS group. NEC-1 application (30 μM, 24 hours) attenuated cell damage induced by H24R4+LPS. n = 4, *P < 0.05 vs control; #P < 0.05 vs H24R4+LPS group.

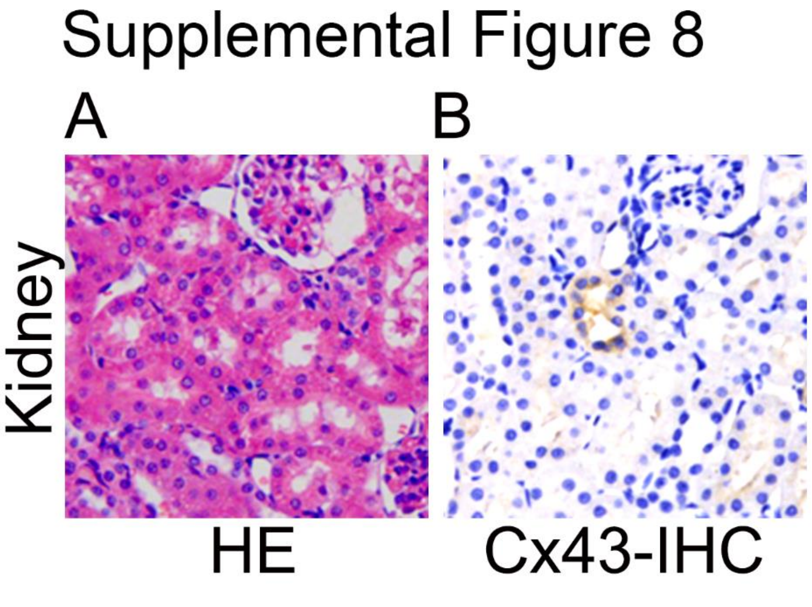

Supplemental Figure 8. Kidney tissues were obtained 2 hours after reperfusion

of patients suffering from kidney transplantation.

(A) Pathological damage (H&E staining; original magnification ×200). (B) Cx43 expression (IHC staining; original magnification ×200). n=6

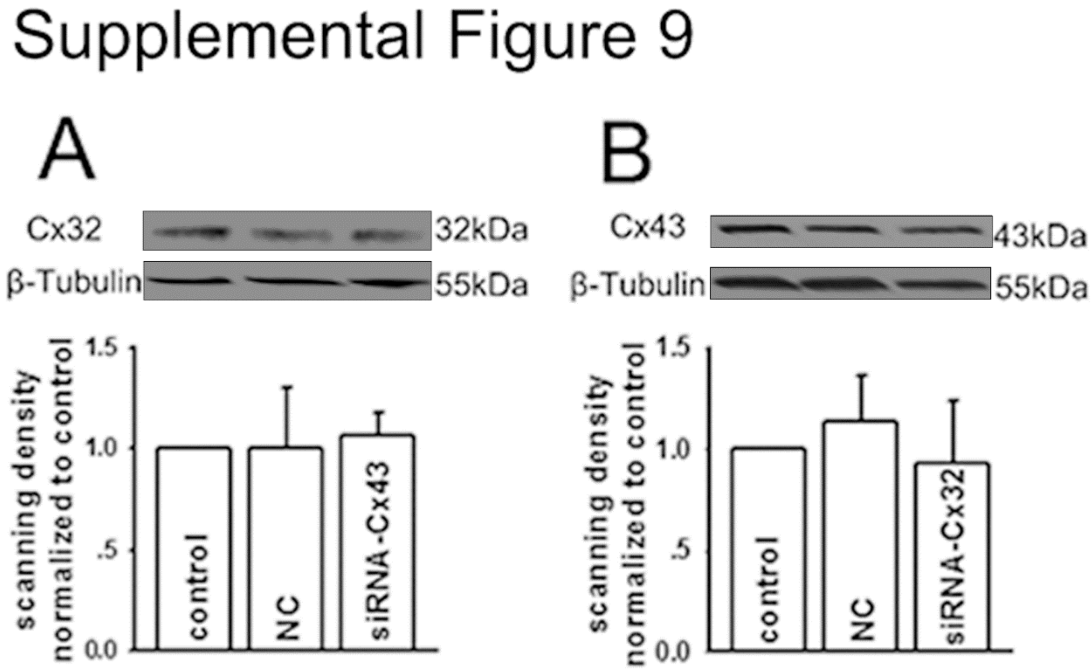

Supplemental Figure 9. The interaction of Cx32 and Cx43 expression in NRK-52E cells.

(A) Cx43 knock-down with siRNA-Cx43 had no effect on Cx32 expression. (B) Cx32 knock-down with siRNA-Cx32 had no effect on Cx43 expression. n = 3.

Supplemental Table 1

The Levels of Rat MAP during AOLT

	MAP (mmHg)	
	Without heptanol	With heptanol
Before operation	99±11.7	96±5.3
Before vascular clamped	94±8.5	99±10.5
Vascular clamped for 1 min	40±7.8	43±7.2
Vascular clamped for 10 min	40±4.2	38±8.8
Vascular clamped for 20min (before vascular unclamped)	44±8.9	39±6.7
Vascular unclamped for 1 min	77±7.9	79±11.4
Vascular unclamped for 10 min	87±7.4	81±8.5
Vascular unclamped for 20 min	89±10.3	94±10.2
Before operation finished	104±7.8	99±12.5
After operation for 1 min	100±11.7	106±8.6
After operation for 10 min	109±9.3	101±11.3

Changes of rat MAP during AOLT. Data are mean ± SEM, n = 8 per group. AOLT = autologous orthotopic liver transplantation; MAP = mean artery blood pressure.
